# 2D printed multicellular devices performing digital and analogue computation

**DOI:** 10.1038/s41467-021-21967-x

**Published:** 2021-03-15

**Authors:** Sira Mogas-Díez, Eva Gonzalez-Flo, Javier Macía

**Affiliations:** Synthetic Biology for Biomedical Applications Lab, Department of Experimental and Health Sciences. Universitat Pompeu Fabra, Biomedical Research Park, Barcelona, Spain

**Keywords:** Assay systems, Synthetic biology

## Abstract

Much effort has been expended on building cellular computational devices for different applications. Despite the significant advances, there are still several addressable restraints to achieve the necessary technological transference. These improvements will ease the development of end-user applications working out of the lab. In this study, we propose a methodology for the construction of printable cellular devices, digital or analogue, for different purposes. These printable devices are designed to work in a 2D surface, in which the circuit information is encoded in the concentration of a biological signal, the so-called carrying signal. This signal diffuses through the 2D surface and thereby interacts with different device components. These components are distributed in a specific spatial arrangement and perform the computation by modulating the level of the carrying signal in response to external inputs, determining the final output. For experimental validation, 2D cellular circuits are printed on a paper surface by using a set of cellular inks. As a proof-of-principle, we have printed and analysed both digital and analogue circuits using the same set of cellular inks but with different spatial topologies. The proposed methodology can open the door to a feasible and reliable industrial production of cellular circuits for multiple applications.

## Abstract (web summary)

Synthetic biology circuits are finding application in a wide range of computational devices, such as contaminant detection. Here, the authors design 2D paper circuits in which the spatial orientation of the cellular components specifies function.

## Introduction

To date, most cellular computational devices have been designed according to a standard architecture that organizes their components into three different layers: the input layer, where external signals are detected; the processing layer, where computation is performed; and the output layer, where the final output is produced^1–4^. However, when this architecture is applied to biological substrates^3,5^, different constraints such as the so-called wiring problem^6–8^, the genetic complexity^8–10^ or the metabolic burden^11–14^ limit the development of complex devices based on this architecture and these limitations^15–17^ can compromise the development of end-user applications^18–20^. Although there are some studies proposing optimal methodologies and software solutions to facilitate complex circuit designs inspired on electronic design automation (EDA)^21,22^, issues regarding scalability, reusability, engineering complexity and technological transference remain a challenge. In previous works, the idea of multicellularity has been applied to design complex computational circuit^23^. However, co-culturing different cell strains has some limitations when considering temporal stability, easy experimental setup and reproducibility for potential applications.

In this study, we explore a framework for computational devices based on biological substrates completely different from the standard architecture, as no distinctions between input and processing layers are considered. By breaking with the conventional architecture and introducing the space^24^ as an additional computational element^25,26^, we demonstrate that simple, robust and scalable multicellular computational devices can be built. This methodology, inspired from printed electronics^27–31^, has enabled us to construct 2D printable cellular devices capable to undertake any type of computation, either digital or analogic. These circuits can be printed in flexible substrates such as paper, allowing the creation of versatile devices. For example, complex single-use living biosensors can be industrially produced at a low cost with potential applications in different market segments.

## Results

### Circuits design

In this study, two main issues needed to be addressed for the development of 2D printed circuits: (i) the optimal circuit architecture and (ii) the methodology for fast and reliable printing.

Regarding the first issue, we propose a multicellular architecture. Considering that computation is, in essence, a matter of information processing^32^, our approach encodes the information in the concentration of a unique biological signal, the carrying signal (CS)^33,34^.

We constructed our devices in a 2D surface through which the CS, which is produced at a specific location in the device, can diffuse. While diffusing, the CS can interact with the different device components which are distributed on the surface in a specific spatial arrangement. The main role of these components is to modulate the flow of the CS in one of two different ways: these can either allow, or even amplify the flow of the CS across them, or can reduce or abolish the CS flow. When these modulatory elements act by only allowing or blocking the CS flow, the device performs digital computations. On the other hand, when regulatory elements increase or reduce the concentration of the CS, and these variations are meaningful in terms of output production, the device performs analogic computations.

The ability of codifying the computational complexity in the topology of modulatory elements, i.e. in their specific spatial arrangement, enables the construction of systems that have very low genetic complexity but that are capable of implementing complex computations^6^. It should be noted that in these devices, the spatial sequence in which the CS meets the different modulatory elements defines the computation. Thus, the same modulatory elements can implement different computations by just altering their spatial position.

The use of a multicellular implementation for computational devices has been shown to be very useful for complex circuits^23,24,26,35^ as each component is implemented in a different cell type. Moreover, this embodiment is appealing because it simplifies the genetic engineering required, thereby minimizing the emergence of unexpected interactions with the host cell and significantly reducing the metabolic burden associated with foreign genes^9,36^. Furthermore, the growth of different cell types in different spatial locations provides devices with high stability since negative competition effects^37,38^, which emerge when multiple cell types grow in the same media, are avoided.

### Development of a library of engineered cells

In order to experimentally validate our approach, we built a library of engineered cells. The genetic architecture of each cell type is schematically shown in Supplementary Fig. 1 and each specific genetic part is summarized in Supplementary Table 1 (see Supplementary Information for details). First, four cell types, S_1_, S_2_, S_3_ and S_4_ were engineered to produce constitutively (S_1_), or upon external induction (S_2_ upon arabinose, S_3_ upon rhamnose and S_4_ upon mercury), a small extracellular molecule that would act as the CS. This molecule is the bacterial 3OC6HSL acyl homoserine lactone (AHL) which is involved in the natural quorum sensing mechanism^39,40^. AHL molecules can diffuse along the paper surface and thereby encounter the different modulatory elements. In our design, unlike in standard methods, there are no explicit connections between the different components of the circuit. Instead, each component contributes independently to the final computation by partially modifying the AHL flow. In consequence, this approach overcomes the so-called wiring problem.

Regarding the modulatory elements, these elements were implemented in differently engineered cell types that express, or do not express Aiia, an enzyme that degrades AHL^41,42^, in response to external inputs spread over the whole device surface. More specifically, some modulators (M^−^ cells) reduce or even abolish the flow of AHL by inducing Aiia expression upon the presence of an external inducer. In contrast, in other modulators (M^+^ cells), Aiia is constitutively expressed and external inducers block this constitutive expression, thereby increasing the flow of AHL. Finally, to compensate the AHL concentration decrease associated with the diffusion process, an auto-amplifier element (CA) was also created. This auto-amplifier cell can produce AHL only when AHL is detected and in this way, restores the AHL signal.

The final overall circuit response was embodied in a specific cell type (reporter cell, CR) that produces the output only when the CS concentration exceeds a given threshold. As a proof-of-principle we expressed a green fluorescent reporter protein (GFP) as the final output. Supplementary Fig. 2 shows the relationship between AHL concentration and GFP expression. It should be noted that each cell type also constitutively expresses red fluorescent protein (RFP) in order to quantitatively assess the cell population growth on the surface. Supplementary Fig. 3 shows the temporal change in RFP expression associated with cell growth in the various cell types (see Supplementary Information for details regarding experimental measurements and quantifications). Experimental results indicate similar dynamic growths for the different cell types.

### Circuit substrates

Despite there being several suitable materials that could act as the working substrate, paper was selected due to its low cost and its ability to allow bacterial growth and AHL diffusion. It is worth mentioning that previous studies have also used paper for different cell-based applications or organic transistors due to these optimal properties^43–45^. To take full advantage of our approach, we also developed a set of cellular inks composed of a single cell type mixed with cellular nutrients and agar (used as a thickener). These inks were used to draw the different circuits in the paper (see Supplementary Information for details regarding the cellular inks and paper used).

### Printing devices

The second issue addressed was the methodology for circuit printing. In this study, we explored the construction of different cellular devices by stamping them on paper surfaces. For it, we used a container filled with different cellular inks. Then, a stamping template was soaked with the different cellular inks and stamped on a paper surface according to a given topology, codifying the computation to be performed. Finally, the stamped paper was deposited on an LB-agar plate mixed with the different inputs, and it was subsequently incubated at 37 °C for 24 h. Stamping offers numerous advantages over previous methods for cell printing such as 3D printers^46^. For instance, stamping is faster than 3D printing, does not require complex printing devices and templates can be easily customized, which makes stamping suitable for industrial production potentially at very low cost and with minimal requirements. Supplementary Fig. 4 shows a schematic representation of the stamping pipeline.

To endorse the proposed methodology, we first analysed cell deposition and cell growth on a paper substrate, as well as CS transmission along the surface. Supplementary Fig. 5a shows a printed pattern composed of a constitutive AHL supply (S_1_) at one end of the strip and reporter cells (CR) uniformly distributed along the rest of the surface. Quantification of the GFP produced in the reporter cells indicates that S_1_ cells can grow in the surface and secrete AHL, which efficiently diffuses along the paper and can be detected by the CR cells at large distances, i.e. up to 20 mm away from the AHL cell source. The ability of AHL auto-amplification by CA cells, printed between S_1_ and CR cells, to restore the signal decay due to diffusion is also analysed in Supplementary Fig. 5b. Experimental results demonstrate that CA cells can extend the range of the diffusing CS.

### Biosensor prototype responding to mercury

In order to prove potential applications of paper-based cellular devices, we created a very simple paper-based biosensor prototype responding to mercury. This device is based on the minimal architecture shown in Supplementary Fig. 5a, composed by a CS supply cell (S_4_) that secretes AHL in the presence of mercury combined with a reporter cell CR expressing GFP in response to AHL (see Supplementary Fig. 1 and Supplementary Table 2 for details about the genetic architecture of the involved cells). CR cells have been distributed in dots along the paper strip. Depending on the mercury concentration, different amounts of AHL are produced, i.e. higher AHL production implies larger AHL diffusion. In consequence, the number of dots expressing GFP (green dots) correlates with the mercury concentration and can visually be estimated. Experimental results are shown in Supplementary Fig. 6. Supplementary Fig. 6a shows the correlation between mercury concentration and number of green dots observed. Supplementary Fig. 6b shows photographs of the visual readout at different mercury concentrations. Results demonstrate a responsive range between 0.013 and 2.71 ppm. It is established that the allowed amount of mercury in most commercialized products is between 0.1 and 1 ppm. Thus, this prototype could be optimal for a fast and easy product screening to ensure safety.

### Transistor-like devices

**Fig. 1:**
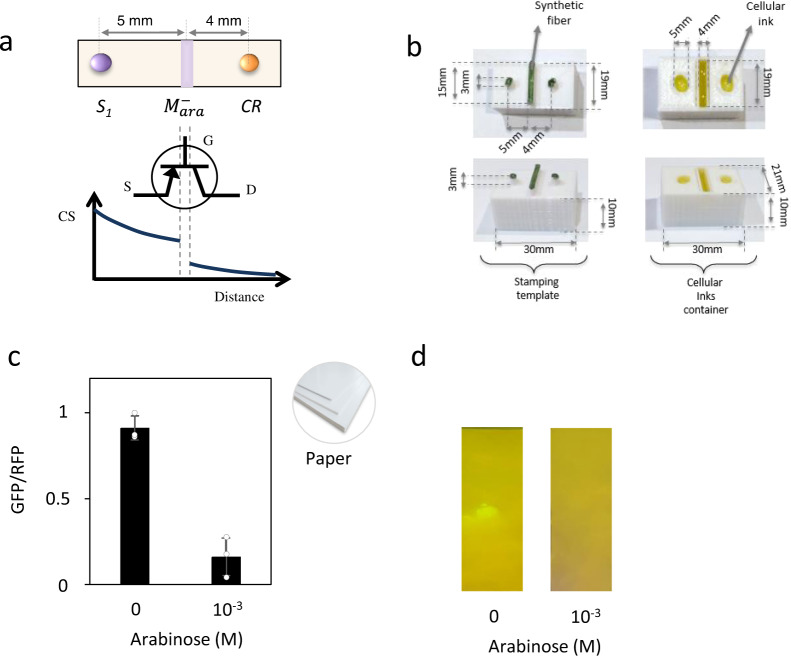
Printed circuit implementing a transistor-like device. **a** Mapping of a general transistor architecture on a cellular printed pattern obtained using a stamping template. Similar to the transistor architecture, the cellular pattern is composed of three main components: source (S_1_ cells), gate (M^−^ cells) that responds to external inputs and a drain (CR cells) as the final output responding to the presence of the carrying signal (CS). **b** Stamping template used to create the circuit made of PLA with a layer of synthetic fibre (green). Cellular inks (yellow) are in their corresponding containers. Before stamping, the synthetic fibre is soaked with the different cell types. Finally, the stamping template is pressed against the paper surface, depositing all cells. **c** Circuit response. In the absence of external input, i.e. arabinose, the CS encoded in the production of AHL molecules by S_1_ cells diffuses along the surface, inducing GFP expression in reporter cells CR. In the presence of 10^−3^ M arabinose (Ara), the modulatory element M^−^_ara_ produces the AHL cleaving enzyme Aiia, which degrades the CS. Error bars are the standard deviation (SD) of three independent experiments. Data are presented as mean values ± SD. Experiments are performed on paper strips. The average fold change is 5.6x. **d** Photography of the device. Source data are provided as a Source Data file.

To implement more complex devices, we analysed the effect of CS modulation on the final output by printing different modulatory elements between S_1_ and CR. The resulting circuits can be compared to electronic transistor architecture, in which the CS, i.e. AHL molecules, enter into the device (source), are modulated in response to an external signal (gate) and then exit the device (drain)^47^. Figure 1 shows an example of a transistor-like circuit where all components can be directly mapped onto our circuit topology. Circuits were assembled by stamping cells using the template shown in Fig. 1b. This template was created in polylactic acid plastic (PLA) with a 3D printer combined with a synthetic fibre tissue (80% polyester and 20% polyamide) in which cells are embedded. Figure 1c shows the behaviour of the device. In the absence of the external input, i.e. arabinose, the CS (AHL molecules) is constitutively produced by S_1_ cells and diffuses along the surface, activating GFP production by the CR reporter cells. In the presence of arabinose (10^-3^ M) the modulatory element${\mathrm{M}}_{{\mathrm{ara}}}^ -$, inserted between the S1 and CR cells, produces Aiia, which degrades AHL, thereby preventing further AHL flow.

Although this study focuses on the use of paper as a substrate, other different materials can be used for printed circuits. As a proof-of-principle, we have printed the same circuit shown in Fig. 1a on nylon fabric. As seen in Supplementary Fig. 7, the circuit printed on nylon has also a good performance.

It is worth mentioning that printed circuits can be either used directly to perform the desired computation or can be stored, in the fridge at 4 °C or frozen at −80 °C, for future use. To evaluate the temporal stability of the transistor-like circuit described in Fig. 1, we analysed the performance evolution of this printed circuit along time, storing them at 4 °C for several days. Experimental results, shown in Supplementary Fig. 8, indicate that circuits stored at 4 °C exhibit a progressive reduction of the fold-change over time, i.e. up to 10 days. Despite this reduction, the device exhibits a proper behaviour. On the other hand, frozen circuits do not exhibit significant changes in their performance, maintaining an optimal functionality, leading to larger durability.

**Fig. 2:**
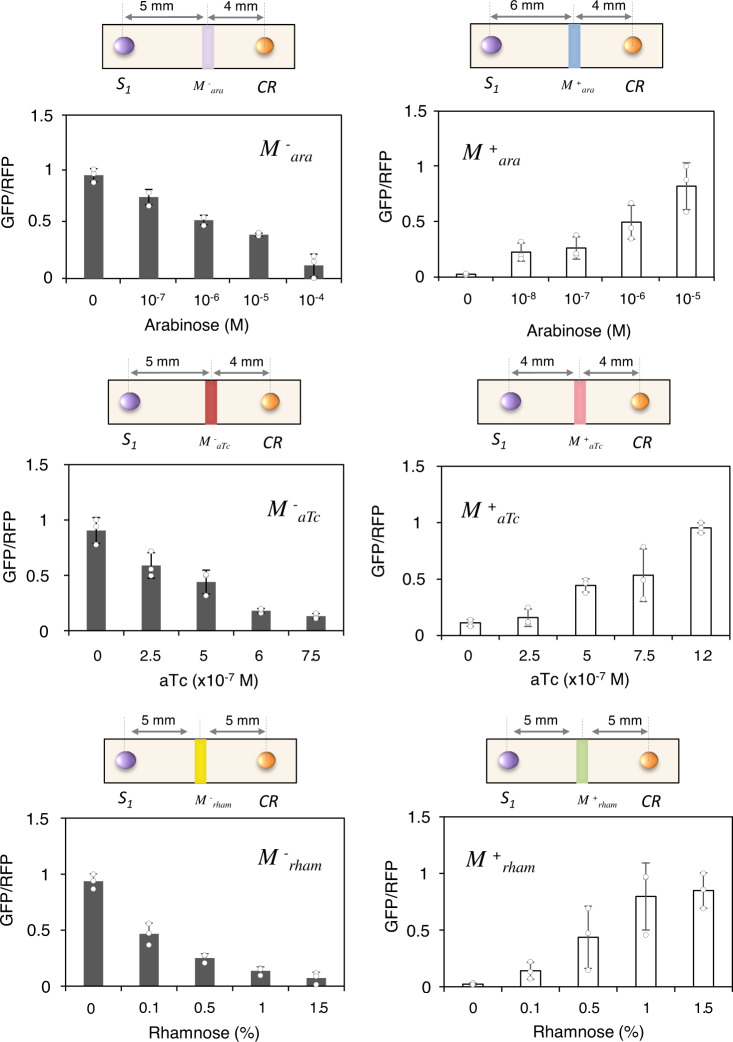
Relationship between input and output in transistor-like circuits. Output modulation in response to different input concentrations. Modulatory elements M^−^ (black bars) and M^+^ (white bars) cells respond to the external inputs, arabinose (M^+^_ara_/M^−^_ara_), aTc (M^+^_aTc_/M^−^_aTc_) and rhamnose (M^+^_rham_/M^−^_rham_). S_1_ cells produce AHL constitutively. GFP levels are normalized by RFP, which correlates with cell population. CR are the reporter cells. Error bars are the standard deviation (SD) of three independent experiments. Data are presented as mean values ± SD. Source data are provided as a Source Data file.

From this first transistor-like architecture, we analysed the CS modulation. Specifically, we tested six different modulatory elements that respond to one of two external signals, aTc, arabinose and rhamnose. Three of the modulatory elements (M^+^_aTc_, M^+^_ara_ and M^+^_rham_ cells) allow the CS flow across the circuit when the level of the external signal increases, whereas the other three modulatory elements (M^−^_aTc_, M^−^_ara_ and M^−^_rham_ cells) reduce the CS flow when the level of the external signal decreases. The experimental results shown in Fig. 2 demonstrate that these modulations are, in essence, analogue, allowing the tuning of CS levels in a continuous manner. This figure displays the output data relative to cell population, i.e. ratio of GFP/RFP. In consequence, by properly combining these modulatory elements, either analogue or digital circuits can be implemented.

Previous to the creation of more complex computational devices, the effect of introducing multiple modulatory elements between S_1_ and CR cells was analysed. In absence of modulatory elements, the optimal distance between S_1_ and CR cells is 10 mm. However, as Supplementary Fig. 9 shows, when an increasing number of modulatory elements are introduced between S_1_ and CR cells, a reduction of the CS levels is observed due to their basal activity. However, this reduction can be restored by introducing CA cells, which allow the introduction of more modulatory elements, overcoming this limitation.

### Development of printed digital circuits

**Fig. 3:**
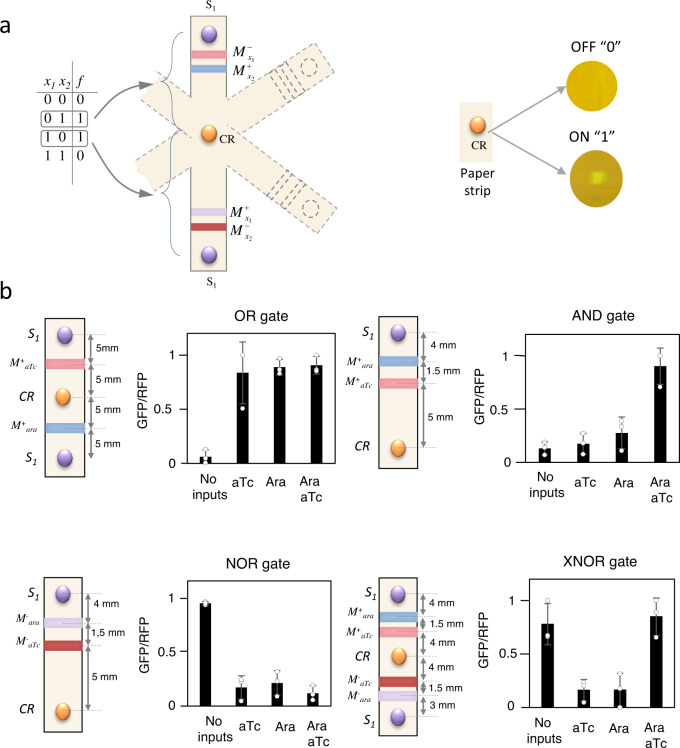
General multi-branch architecture for implementation of digital circuits. **a** Schematic representation of the multi-branch implementation of a truth table. **b** Implementation of different logic gates. A schematic representation of the cells used in each paper strip and their corresponding distance points is given (Left). Gates with two sources of *S*_*1*_ (OR and XNOR gates) are circuits carrying two branches, while the other gates (NOR and AND gates) can be implemented with just one branch. Input concentrations are Ara = 10^−3^ M and aTc = 10^−6^ M. M^+^_aTc_ and M^−^_aTc_ are, respectively, positive and negative modulatory cells responding to aTc. M^+^_ara_ and M^−^_ara_ are, respectively, positive and negative modulatory cells responding to arabinose. S_1_ cells produce AHL constitutively and CR are the reporter cells. Error bars are the standard deviation (SD) of three independent experiments. The average fold change has been obtained from the mean of ON and OFF states from each circuit. OR gate 14.31x, AND gate 6.21x, NOR gate 6.58x, XNOR gate 5.6x. Source data are provided as a Source Data file.

To develop printed digital circuits, we explored a general method for mapping any arbitrary truth table into a spatial pattern. Specifically, we proposed the multi-branch circuit that is schematically represented in Fig. 3a. In this topology, each input combination in a truth table associated with output 1 can be encoded in a different branch. At one end of the branch, there are AHL producer cells, whereas at the other end, there is the output, produced by CR cells. In the middle, different bands containing modulatory cells are printed. Logic inputs “1” in the truth table are encoded in M^+^ cell types, which allow CS diffusion only if the input is present. Logic inputs “0” are encoded in M^−^ cell types, which allow CS diffusion only when the input is absent. We analysed the scalability of this topological implementation. For circuits responding to *N* inputs, the maximum size of the cell library implementing modulatory elements is 2·*N*, i.e. two types of modulatory element, M^+^ and M^−^, for each input. Moreover, the maximum number of branches is 2 ^*N*-1^. However, our computational analysis, shown in Supplementary Fig. 10, indicates that most of the circuits require less branches. To perform this analysis, the truth tables of all circuits responding to 2 (Supplementary Fig. 10a), 3 (Supplementary Fig. 10b) and 4 inputs (Supplementary Fig. 10c) were simplified to its minimal configuration using the standard Karnaugh maps method^48^ and directly mapped into a topological configuration, according to the method represented in Fig. 3a.

These results indicate that large scalability is achievable with this multi-branched architecture. As a proof-of-principle, we implemented several examples of logic gates using the multi-branch architecture. Relative GFP/RFP values obtained from these examples are shown in Fig. 3b.

**Fig. 4:**
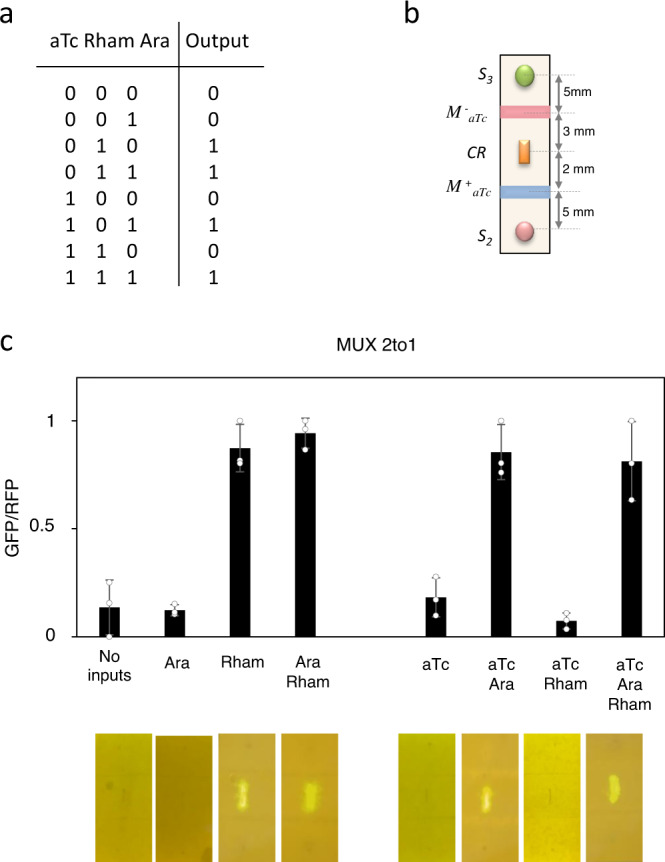
Multiplexor 2 to 1. **a** Truth table of the 2 to 1 multiplexer. **b** Spatial pattern of cells encoding the multiplexer device with their corresponding distances. M^+^_aTc_ and M^−^_aTc_ are, respectively, positive and negative modulatory cells responding to aTc. S_2_ cells produce AHL upon arabinose induction and S_3_ cells produce AHL upon rhamnose induction. CR are the reporter cells. **c** Multiplexer response upon input of the indicated combinations. Input concentrations are Ara = 10^−3^ M, aTc = 10^−6^ M and Rham = 1.5%. Error bars are the standard deviation (SD) of three independent experiments. Data are presented as mean values ± SD. The average fold change has been obtained from the mean of ON and OFF states from each input combination. Multiplexor 2 to 1 fold change: 6.76x. Source data are provided as a Source Data file.

Once logic gates responding to two inputs were constructed, we introduced an additional input, i.e. rhamnose, in order to create a 3-input device. As a proof-of-concept, we implanted a 2 to 1 multiplexer (MUX2to1). This device involves two branches producing AHL by S_3_ and S_2_ cells in response to rhamnose and arabinose, and different modulatory elements responding to aTc. Figure 4a shows the truth table describing the behaviour of this device and Fig. 4b displays the specific spatial topology. Figure 4c shows the circuit response upon different input combinations. As seen in this figure, the device behaves according to the truth table, implementing the expected response.

**Fig. 5:**
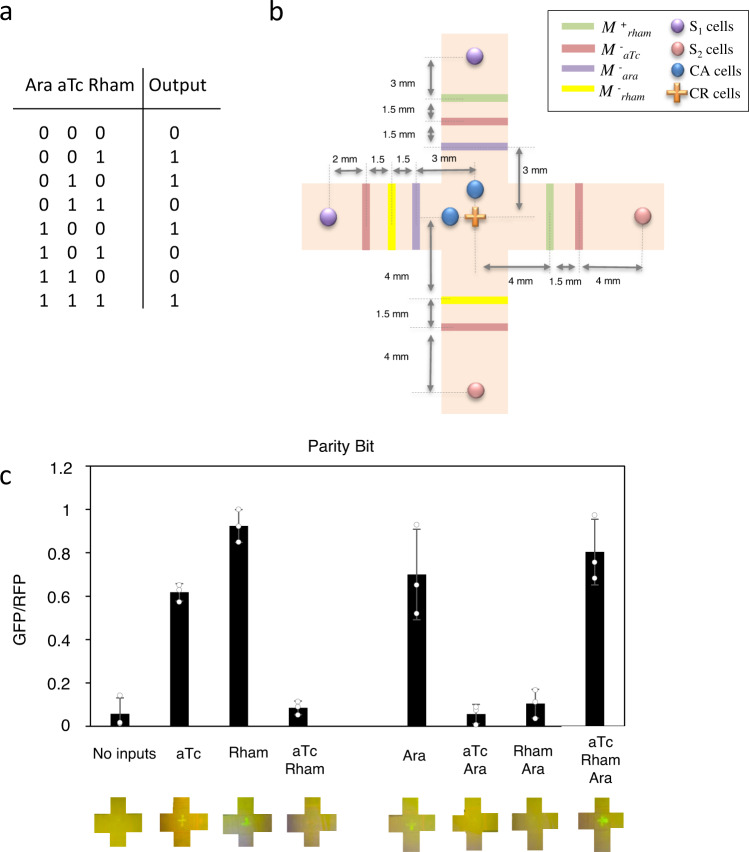
Parity bit. **a** Truth table of the even parity bit circuit. **b** Spatial pattern of the cells encoding the truth table. This circuit involves four branches, with the final output in the middle. The output cells CR were printed as a cross to visualize from which branch comes dominantly the AHL signal. M^+^_aTc_ and M^−^_aTc_ are, respectively, positive and negative modulatory cells responding to aTc. M^+^_rham_ and M^−^_rham_ are, respectively, positive and negative modulatory cells responding to rhamnose. S_1_ cells produce AHL constitutively and S_2_ cells produce AHL upon arabinose induction. **c** Multiplexer response upon input of the indicated combinations. In this circuit, due to the amount of modulatory elements per branch, auto-amplifier cells (CA) were incorporated close to reporter cells (CR). These cells amplify the signal on the presence of AHL. Input concentrations are ara = 10^−3^ M, aTc = 10^−6^ M and rham = 1.5%. Error bars are the standard deviation (SD) of three independent experiments. Data are presented as mean values ± SD. The average fold change has been obtained from the mean of ON and OFF states from each input combination. Parity bit fold change: 10.47x Source data are provided as a Source Data file.

To explore more complex topologies, an even parity bit circuit was implemented. This circuit, responding to three different inputs, involves four different branches with up to three modulatory elements per branch. Due to this large number of modulators, an auto-amplifier cell (CA) was introduced close to the output cells CR in order to restore AHL levels. Figure 5a shows the truth table describing circuit’s logics, which is translated into a printed pattern (Fig. 5b). Experimental results are shown in Fig. 5c, indicating a proper circuit response.

### Development of analogic circuits

**Fig. 6:**
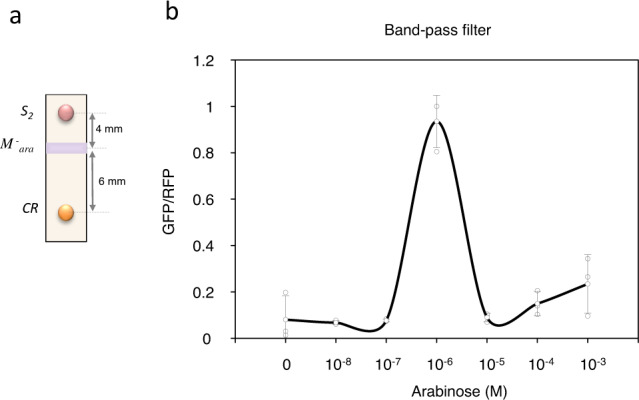
Band-pass filter circuit. **a** Band-pass filter implementation is outlined by a schematic representation of the cells used for the paper strips and their corresponding distance points. M^−^_ara_ are negative modulatory cells responding to arabinose. S_2_ cells produce AHL upon arabinose induction and CR are the reporter cells. **b** Circuit response upon increasing concentrations of the external input, i.e. arabinose. Error bars are the standard deviation (SD) of three independent experiments. Data are presented as mean values ± SD. Source data are provided as a Source Data file.

Not only digital, but also analogic computations were considered to prove the versatility of printed circuits. For it, an example of an analogic circuit, i.e. a band-pass filter, was implemented using the same elements described in Fig. 1, except that cell type S_1_ (constitutive production of AHL) was replaced by S_2_ (arabinose-inducible production of AHL). This allows the introduction of an additional modulatory element as AHL production depends on the external input, in this case arabinose. At low arabinose concentrations, AHL levels are low due to the low production in S_2_ cells. The combination of low AHL synthesis with the reduction of AHL due to its diffusion does not allow AHL-induced output production by CR cells. In contrast, at high arabinose concentrations, although the higher AHL secretion, it also induces the production of Aiia by the modulatory element (M^−^_ara_ cells) located between S_2_ and CR cells, which degrades the diffusing signal and therefore, blocks the AHL flow and the output is not produced. However, at intermediate arabinose concentrations, the level of AHL secreted from S_2_ cells is sufficient for CR activation but the arabinose concentration is not high enough for M^−^_ara_ activation. Thus, AHL concentration induces output production in CR cells. Figure 6 shows the GFP/RFP levels at different arabinose concentrations, indicating a clear band-pass response.

## Discussion

Multiple studies have demonstrated that multicellular embodiments are optimal to implement complex computational devices. However, co-culturing different cell types in the same medium induces the emergence of problems related to competition for nutrients, which can compromise large temporal stability. This problem has been overcome introducing spatial segregation^24^. However, in general, this methodology requires multiple wiring molecules coexisting, which can limit the scalability. Other approaches only require a single wiring molecule but the experimental setup is complex and the composition and stability of the multicellular consortia must be carefully controlled^23^. The methodology presented in this study combines the benefits of the previous approaches but overcoming some of their limitations, namely only a single molecule is required for computation and different cell types can growth separately, avoiding competition effects.

Although other approaches based on cell-free systems^49^ are suitable for paper-based devices, the limited number of biochemical reactions available in these systems could limit the creation of complex devices involving multiple circuit components. The present results are just a small piece of the potentially far-reaching implications and possibilities of this approximation. For instance, it has been demonstrated that very simple but useful biosensors can be easily implemented in paper-based circuits (e.g. mercury detector shown in Supplementary Fig. 6) and used out of the lab, allowing a rapid estimation of mercury concentration directly from the visual reporter. Additionally, more complex devices able to detect and integrate different signals to produce the output only upon certain combination have also been built.

Although our system works for three inputs, future work should be devoted to (i) scaling up this system to enlarge the possible computations, (ii) automatize the stamping of the complex circuits for large-scale industrial production and (iii) reduce time response.

In summary, printable cellular circuits could represent an interesting alternative for paper-based devices with appealing potential advantages such as the possibility of producing low-cost cellular devices based on a simple experimental setup methodology. Further development is required to explore this potential and meet further market needs.

## Methods

### Strains, media and growth conditions

Cloning and expression experiments were performed in Escherichia Coli Top10 (Invitrogen, USA) and Escherichia Coli ZN1 (ExpresSys, Germany). Cells were grown in Lysogeny Broth (LB) at 37 ^◦^C and selected with appropriate antibiotics (chloramphenicol 35 µg/ml, kanamycin 35 µg/ml Sigma, USA). Bacterial strains were preserved in LB glycerol 20% (v/v) at −80^◦^C. Cells inoculated from single colonies from streaked glycerol stocks were grown at 37 °C with shaking (450 revolutions per minute (rpm)) for about 5 h until reaching an OD between 0.15–0.2.

Experiments were performed in petri dishes (ddbiolab) pouring 20 ml of LB broth with agar (Sigma Aldrich, USA) supplemented with the antibiotic and the appropriate inducers before depositing the paper strips to print the circuit.

The inducers used were L^−^( ^+^ )-arabinose 98% (Sigma Aldrich, USA), N-3-oxo-tetradecanoyl-L-Homoserine lactone (Cayman Chemical, USA) and Anhydrotetracycline 98% (Cayman Chemical,USA).

### Cellular inks

Cellular inks were elaborated by preparing a cell culture from single colonies obtained from streaked glycerol stocks and grown until reaching an optical density (OD) of 0.2. From these cultures, two different cellular inks were elaborated, one for ready-to-use circuits and another one to have circuits stored at −80 °C. For the first ones, these cell cultures were mixed with LB agar, prepared by adding 0.6 g of agar to 50 mL of LB and boiling it until having everything diluted. After cooling to ~50 °C properly, antibiotic selection and cell cultures were added to obtain each cellular ink. The use of agar in the mixture prevents cell culture dispersion when deposited on paper surface, providing some cellular confinement and to have a better control of the positioning of each cellular element. For circuits stored at −80 °C, the same principles were used but, in this case, a 20% v/v glycerol was used for the mixture, rather than the agar. Glycerol cryoprotects cells deposited on paper, so circuits can be thawed and used later.

More specifically, for the modulatory elements, a volume of 100 µl of cell culture was mixed with 50 µl of liquid LB-agar mixture. For the rest of elements, i.e. the S_1_, S_2_, CA and CR cells, a volume of 50 µl of cell culture was mixed with 50 µl of LB-agar, also in liquid state.

### Paper properties

The paper used for all experiments was 75 gsm White Paper (Pack of 2500) 59908. It has an optimal quality due to its eucalipto globulus fibres mixed with calcium carbonate. This combination allows to produce a 75 g/m^2^ paper with necessary rigidity and opacity.

### Cell growth

Because cell population grown in a paper surface cannot be directly estimated by optical density measures, all cell strains constitutively express RFP. We assume that cell population has a linear relationship with RFP levels as RFP expression at single-cell level is at the steady state. Supplementary Fig. 2 shows the temporal evolution of RFP levels associated to cell growth in a paper surface. Experimental results indicate that the different cell types have similar growth dynamics.

### GFP quantification

Reporter cell CR expresses GFP in response to AHL diffused throughout paper surface. In order to characterize its induction range, several LB plates containing different concentrations of AHL, from 10^−4^ M to 10^−10^ M were prepared. Later, small pieces of paper were placed on the top of each plate and a dot of 1 µl of the reporter cellular ink was added. The plate was grown at 37 °C overnight and after 24 h, a surface scanning was carried out, measuring GFP and RFP intensities. Measures were performed with *Synergy HXT- Hybrid Multi-Mode Reader* (BioTek Instruments, USA). Experimental data were collected with Gen5 software package and analysed with Microsoft Excel 2013. Supplementary Fig. 4 shows the CR response at different AHL concentrations. GFP levels were normalized by RFP that is assumed proportional to cell population. Experimental data was mathematically fitted to a Hill function

1 $${\mathrm{GFP}}/{\mathrm{RFP}} = \alpha _{{\mathrm{AHL}}} + \frac{{k_0\cdot [{\mathrm{AHL}}]^n}}{{k_1 + [{\mathrm{AHL}}]^n}}$$

Fitting parameters are *α*_AHL_ = 1, *k*_0_ = 33 M^−1^, *k*_1_ = 4 × 10^7^ M and *n* = 1. Fluorescent measures for GFP: excitation wavelength: 475 ± 9 nm, emission wavelength: 509 ± 9 nm. Fluorescence measures for RFP: excitation wavelength: 578 ± 9 nm, emission wavelength: 616 ± 9 nm.

### Reporting summary

Further information on research design is available in the Nature Research Reporting Summary linked to this article.

## Supplementary information

Supplementary Information

Reporting Summary

## Data Availability

Plasmids and strains used in this study and other materials are available upon request to authors. Correspondence and requests for materials should be addressed to J.M. (javier.macia@upf.edu). DNA sequences for all constructs are provided as Supplementary Information. Genetic parts used in this work are available at Registry of Standard Biological Parts (http://parts.igem.org/). Source data are provided with this paper.

## Source data

Source Data

## Code availability

Scripts used to analyse the scalability of the circuits are available at https://github.com/jmacia-upf/printed_circuits.git.

## References

[1] Brophy JAN, Voigt CA (2014). Principles of genetic circuit design. Nat. Methods.

[2] Grozinger L (2019). Pathways to cellular supremacy in biocomputing. Nat. Commun..

[3] Slusarczyk AL, Lin A, Weiss R (2012). Foundations for the design and implementation of synthetic genetic circuits. Nat. Rev. Genet..

[4] Kobayashi H (2004). Programmable cells: interfacing natural and engineered gene networks. Proc. Natl Acad. Sci. USA.

[5] Zakeri B, Carr PA (2015). The limits of synthetic biology. Trends Biotechnol..

[6] Karamasioti E, Lormeau C, Stelling J (2017). Computational design of biological circuits: putting parts into context. Mol. Syst. Des. Eng..

[7] Macía J, Posas F, Solé RV (2012). Distributed computation: the new wave of synthetic biology devices. Trends Biotechnol..

[8] Kwok R (2010). Five hard truths for synthetic biology. Nature.

[9] Li B, You L (2011). Division of logic labour. Nature.

[10] Guet CC (2002). Combinatorial synthesis of genetic networks. Science.

[11] Glick BR (1995). Metabolic load and heterologous gene expression. Biotechnol. Adv..

[12] Ceroni F, Algar R, Stan GB, Ellis T (2015). Quantifying cellular capacity identifies gene expression designs with reduced burden. Nat. Methods.

[13] Gyorgy, A. & Del Vecchio, D. Limitations and trade-offs in gene expression due to competition for shared cellular resources. *Proc. IEEE Conf. Decis. Control*, Los Angeles, CA, USA, 2014, pp. 5431–5436, 10.1109/CDC.2014.7040238.

[14] Carbonell-Ballestero M, Garcia-Ramallo E, Montañez R, Rodriguez-Caso C, Macía J (2016). Dealing with the genetic load in bacterial synthetic biology circuits: convergences with the Ohm’s law. Nucleic Acids Res..

[15] Ellis T, Wang X, Collins JJ (2009). Diversity-based, model-guided construction of synthetic gene networks with predicted functions. Nat. Biotechnol..

[16] Sayut DJ, Niu Y, Sun L (2009). Construction and enhancement of a minimal genetic and logic gate. Appl. Environ. Microbiol..

[17] Cardinale S, Arkin AP (2012). Contextualizing context for synthetic biology–identifying causes of failure of synthetic biological systems. Biotechnol. J..

[18] Wang B, Kitney RI, Joly N, Buck M (2011). Engineering modular and orthogonal genetic logic gates for robust digital-like synthetic biology. Nat. Commun..

[19] Sayut DJ, Kambam PKR, Sun L (2007). Engineering and applications of genetic circuits. Mol. Biosyst..

[20] Purnick PEM, Weiss R (2009). The second wave of synthetic biology: from modules to systems. Nat. Rev. Mol. Cell Biol..

[21] Chen Y (2020). Genetic circuit design automation for yeast. Nat. Microbiol.

[22] Nielsen AAK (2016). Genetic circuit design automation. Science.

[23] Macia J (2016). Implementation of complex biological logic circuits using spatially distributed multicellular consortia. PLoS Comput. Biol..

[24] Tamsir A, Tabor JJ, Voigt CA (2011). Robust multicellular computing using genetically encoded NOR gates and chemical ‘wires’. Nature.

[25] Sarpeshkar, R. Analog versus digital: extrapolating from electronics to neurobiology. *Neural Comput.* **10**, 1601–38 (1998). 10.1162/089976698300017052 9744889

[26] Regot S (2011). Distributed biological computation with multicellular engineered networks. Nature.

[27] Khan S, Lorenzelli L, Dahiya RS (2015). Technologies for printing sensors and electronics over large flexible substrates: a review. IEEE Sens. J..

[28] Li Q (2019). Review of printed electrodes for flexible devices. Front. Mater..

[29] Khan Y (2019). A new frontier of printed electronics: flexible hybrid electronics. Adv. Mater..

[30] Tong, G., Jia, Z. & Chang, J. Flexible hybrid electronics: review and challenges. In *Proc. - IEEE Int. Symp. Circuits Syst*. 1–5 (IEEE, 2018).

[31] Kramer BP, Fischer C, Fussenegger M (2004). BioLogic gates enable logical transcription control in mammalian cells. Biotechnol. Bioeng..

[32] Smith, B. C. The Foundations of Computing, in *Computationalism: New Directions* (ed. Scheutz, M.) pp. 23–58. (Cambridge, MA: MIT Press, 2002).

[33] Basu S, Gerchman Y, Collins CH, Arnold FH, Weiss R (2005). A synthetic multicellular system for programmed pattern formation. Nature.

[34] Waters CM, Bassler BL (2005). QUORUM SENSING: cell-to-cell communication in bacteria. Annu. Rev. Cell Dev. Biol..

[35] Guiziou S, Mayonove P, Bonnet J (2019). Hierarchical composition of reliable recombinase logic devices. Nat. Commun..

[36] Oyarzún DA, Stan G-BV (2013). Synthetic gene circuits for metabolic control: design trade-offs and constraints. J. R. Soc. Interface.

[37] Stubbendieck RM, Straight PD (2016). Multifaceted interfaces of bacterial competition. J. Bacteriol..

[38] Khare A, Tavazoie S (2015). Multifactorial competition and resistance in a two-species bacterial system. PLOS Genet..

[39] Wai-Leung NG, Bassler B (2015). Bacterial quorum-sensing network architectures. Annu. Rev. Genet..

[40] Fuqua C, Winans SC, Greenberg EP (1996). CENSUS AND CONSENSUS IN BACTERIAL ECOSYSTEMS: the LuxR-LuxI family of quorum-sensing transcriptional regulators. Annu. Rev. Microbiol..

[41] Lee SJ (2002). Genes encoding the *N*-acyl homoserine lactone-degrading enzyme are widespread in many subspecies of *Bacillus thuringiensis*. Appl. Environ. Microbiol..

[42] Chen F, Gao Y, Chen X, Yu Z, Li X (2013). Quorum quenching enzymes and their application in degrading signal molecules to block quorum sensing-dependent infection. Int. J. Mol. Sci..

[43] Zschieschang U, Klauk H (2019). Organic transistors on paper: a brief review. J. Mater. Chem. C..

[44] Shin H (2019). Highly stable organic transistors on paper enabled by a simple and universal surface planarization method. Adv. Mater. Interfaces.

[45] Peng B, Chan PKL (2014). Flexible organic transistors on standard printing paper and memory properties induced by floated gate electrode. Org. Electron..

[46] Schaffner, M., Rühs, P. A., Coulter, F., Kilcher, S. & Studart, A. R. 3D printing of bacteria into functional complex materials. *Sci. Adv*. **3**, eaao6804 (2017). 10.1126/sciadv.aao6804 PMC5711516 29214219

[47] Horowitz, P. & Winfield, H. *The Art of Electronics* 2nd edn (Cambridge Univ. Press, 1989).

[48] Karnaugh M (2013). The map method for synthesis of combinational logic circuits. Trans. Am. Inst. Electr. Eng. Part I Commun. Electron..

[49] Pardee K (2014). Paper-based synthetic gene networks. Cell.

