# Supplementary material for: 2D printed multicellular devices performing digital and analogue computation
Source: Nat Commun. 2021 Mar 15;12:1679. doi: 10.1038/s41467-021-21967-x (PMC7961044; doi:10.1038/s41467-021-21967-x)
Supplement: Supplementary file 1 — Supplementary Information [file 41467_2021_21967_MOESM1_ESM.pdf]

Supplementary Information

2D printing of multicellular devices: spatial computation for digital and analogue circuits

Sira Mogas-Díez[1], Eva Gonzalez-Flo[1], Javier Macía[1] *

[1] Synthetic Biology for Biomedical Applications Lab, Department of Experimental and Health Sciences. Universitat Pompeu Fabra , Biomedical Research Park, Barcelona, Spain.

*To whom correspondence should be addressed. Tel: +34 933161903; Email: javier.macia@upf.edu

Supplementary Notes

Engineered cells library

The genetic architecture of the engineered cells used in this study is summarized in table S1 together with a schematic representation of these constructs, shown in figure S1. There are four groups of cells.

- **CS supplier cells**: These cells produce de AHL molecules, acting as CS, in different locations of the circuit surface.
  - For digital circuits, cell S1 was built to produce AHL molecules in a constitutive manner by expressing LuxI downstream P_{Tet} promoter, acting as constitutive in *E.coli* Top10 strains.
  - For analog circuits, cell S2 allows the production of AHL in response to an external input, i.e. arabinose, locating LuxI, synthase, responsible of AHL synthesis[1], downstream the arabinose-inducible promoter P_{ara}.

- **Modulator cells**: These cells are responsible of the CS modulation expressing Aiia, which degrades AHL. Here there are two subsets of cells.
  - Negative modulators reduce the AHL concentration in presence of external inputs. This effect is achieved expressing downstream an externally inducible promoter. Specifically, M^{-}_{ara} expresses aiiA under the regulation of the arabinose-inducible promoter P_{ara}[2] in Top10 strain, whereas M^{-}_{aTc} expresses aiiA upon P_{Tet} promoter[3], which is constitutively repressed by TetR in Zn1 strain. In the presence of Anhydrotetracycline (aTc) P_{Tet} promoter is depressed and aiiA is expressed.
  - Positive Modulators that allows the AHL diffusion. These cells express LacI repressor under externally inducible promoters, P_{ara} (M^{+}_{ara}) and P_{Tet} (M^{+}_{aTc}). Finally, the P_{Lac} promoter [Elowitz & Leibier, 2000], which is repressed by LacI, regulates aiiA production.

- **Amplifier cell (CA):** In order to compensate AHL decay due to diffusion gradient, this cell produces AHL in presence of AHL. The implemented architecture is based in the Lux system from *vibrio fischeri*[4]. Receptor protein LuxR, which is constitutively expressed, binds to AHL molecules forming an active dimeric complex. This complex triggers the expression of LuxI under the P_{Lux} promoter.

- **Reporter cell (CR):** In order to validate the correct behaviour of the cellular circuits, this cell produces GFP in response to AHL levels. This cell architecture is based on the same Lux system than CA cell but expressing GFP under P_{Lux} promoter, instead of LuxI.

Supplementary Tables

Cell type	Genetic Structure	Strain
**S1**	B0014 + PTET + B0032 + C0061<br>B0014 + J23105 + B0032 + E1010 + B0014	Top10
**S2**	B0014 + I0050 + B0032 + C0061<br>B0014 + J23105 + B0032 + E1010 + B0014	Top10
**S3**	B0014 + K914003 + B0032 + E1010 + B0014<br>B0014 + J23105 + B0032 + E1010 + B0014	Top10
**S4**	B0014 + J23100 + B0034 + K1420004<br>+ B0014 + K346002 + B0033 + E0040<br>B0014 + J23105 + B0032 + E1010 + B0014	Top10
$\mathbf{M^{+}_{aTc}}$	B0014 + R0040 + B0032 + C0060<br>B0014 + J23105 + B0032 + E1010 + B0014	Zn1
$\mathbf{M^{+}_{ara}}$	B0014 + I0050 + B0032 + C0060<br>B0014 + J23105 + B0032 + E1010 + B0014	Top10
$\mathbf{M^{-}_{aTc}}$	B0014 + R0040 + B0032 + C0012 +<br>B0014+ R0011 + B0032 + C0060<br>B0014 + J23105 + B0032 + E1010 + B0014	Zn1
$\mathbf{M^{-}_{ara}}$	B0014 + I0050 + B0032+ C0012 +<br>B0014+ R0011 + B0032 + C0060<br>B0014 + J23105 + B0032 + E1010 + B0014	Top10
**CA**	B0014 + J23100 + B0033 + C0061<br>+ B0014 + R0061 + B0033 + C0061	Top10

	B0014 + J23105 + B0032 + E1010 + B0014	
**CR**	B0014 + J23100 + B0034 + C0061<br>+ B0014 + R0061 + B0033 + E0040<br>B0014 + J23105 + B0032 + E1010 + B0014	Top10

Supplementary Table 1. Genetic parts involved in each engineered cell strain. Genetic parts were obtained from the Registry of Standard Biological Parts (http://parts.igem.org/).

Part	DNA Sequence
**C0061**	atgactataatgataaaaaaatcggatttttggcaattccatcggaggagtataaaggtattctaagt<br>cttcgttatcaagtgtttaagcaaagacttgagtgggacttagttgtagaaaataaccttgaatcagat<br>gagtatgataactcaaatgcagaatatatttatgcttgtgatgatactgaaaatgtaagtggatgctgg<br>cgtttattacctacaacaggtgattatatgctgaaaagtgtttttcctgaattgcttggtcaacagagtg<br>ctcccaaagatcctaatatagtcgaattaagtcgttttgctgtaggtaaaaatagctcaaagataaat<br>aactctgctagtgaaattacaatgaaactatttgaagctatatataaacacgctgttagtcaaggtatt<br>acagaatatgtaacagtaacatcaacagcaatagagcgatttttaaagcgtattaaagttccttgtca<br>tcgtattggagacaaagaaattcatgtattaggtgatactaaatcggttgtattgtctatgcctattaat<br>gaacagtttaaaaaagcagtcttaaatgctgcaaacgacgaaaactacgctttagtagcttaataact<br>ctgatagtgctagtgtagatctc
**C0060**	atgacagtaaagaagctttatttcgtcccagcaggtcgttgtatgttggatcattcgtctgttaatagta<br>cattaacaccaggagaattattagacttaccggtttggtgttatcttttggagactgaagaaggaccta<br>ttttagtagatacaggtatgccagaaagtgcagttaataatgaaggtctttttaacggtacatttgtcg<br>aagggcaggttttaccgaaaatgactgaagaagatagaatcgtgaatattttaaaacgggttggttat<br>gagccggaagaccttctttatattattagttctcacttgcattttgatcatgcaggaggaaatggcgctt<br>ttataaatacaccaatcattgtacagcgtgctgaatatgaggcggcgcagcatagcgaagaatatttg<br>aaagaatgtatattgccgaatttaaactacaaaatcattgaaggtgattatgaagtcgtaccaggagt<br>tcaattattgcatacaccaggccatactccagggcatcaatcgctattaattgagacagaaaaatccg<br>gtcctgtattattaacgattgatgcatcgtatacgaaagagaattttgaaaatgaagtgccatttgcgg<br>gatttgattcagaattagctttatcttcaattaaacgtttaaaagaagtggtgatgaaagagaagccg<br>attgttttctttggacatgatatagagcaggaaaggggatgtaaagtgttccctgaatatatagctgca<br>aacgacgaaaactacgctttagtagcttaataacgctgatagtgctagtgtagatcgc

**R0032**	tcacacaggaaag
**R0033**	tcacacaggac
**R0034**	aaagaggagaaa
**E0040**	atgcgtaaaggagaagaacttttcactggagttgtcccaattcttgttgaattagatggtgatgttaat<br>gggcacaaattttctgtcagtggagagggtgaaggtgatgcaacatacggaaaacttacccttaaat<br>ttatttgcactactggaaaactacctgttccatggccaacacttgtcactactttcggttatggtgttcaa<br>tgctttgcgagatacccagatcatatgaaacagcatgactttttcaagagtgccatgcccgaaggttat<br>gtacaggaaagaactatatttttcaaagatgacgggaactacaagacacgtgctgaagtcaagtttg<br>aaggtgatacccttgttaatagaatcgagttaaaaggtattgattttaaagaagatggaaacattcttg<br>gacacaaattggaatacaactataactcacacaatgtatacatcatggcagacaaacaaaagaatg<br>gaatcaaagttaacttcaaaattagacacaacattgaagatggaagcgttcaactagcagaccatta<br>tcaacaaaatactccaattggcgatggccctgtccttttaccagacaaccattacctgtccacacaatc<br>tgccctttcgaaagatcccaacgaaaagagagaccacatggtccttcttgagtttgtaacagctgctg<br>ggattacacatggcatggatgaactatacaaataataa

**C0012**	atggtgaatgtgaaaccagtaacgttatacgatgtcgcagagtatgccggtgtctcttatcagaccgtttcccgcgtggtgaaccaggccagccacgtttctgcgaaaacgcgggaaaaagtggaagcggcgatggcggagctgaattacattcccaaccgcgtggcacaacaactggcgggcaaacagtcgttgctgattggcgttgccacctccagtctggccctgcacgcgccgtcgcaaattgtcgcggcgattaaatctcgcgccgatcaactgggtgccagcgtggtggtgtcgatggtagaacgaagcggcgtcgaagcctgtaaagcggcggtgcacaatcttctcgcgcaacgcgtcagtgggctgatcattaactatccgctggatgaccaggatgccattgctgtggaagctgcctgcactaatgttccggcgttatttcttgatgtctctgaccagacacccatcaacagtattattttctcccatgaagacggtacgcgactgggcgtggagcatctggtcgcattgggtcaccagcaaatcgcgctgttagcgggcccattaagttctgtctcggcgcgtctgcgtctggctggctggcataaatatctcactcgcaatcaaattcagccgatagcggaacgggaaggcgactggagtgccatgtccggttttcaacaaaccatgcaaatgctgaatgagggcatcgttcccactgcgatgctggttgccaacgatcagatggcgctgggcgcaatgcgcgccattaccgagtccgggctgcgcgttggtgcggatatctcggtagtgggatacgacgataccgaagacagctcatgttatatcccgccgttaaccaccatcaaacaggattttcgcctgctggggcaaaccagcgtggaccgcttgctgcaactctctcagggccaggcggtgaagggcaatcagctgttgcccgtctcactggtgaaaagaaaaaccaccctggcgcccaatacgcaaaccgcctctccccgcgcgttggccgattcattaatgcagctggcacgacaggtttcccgactggaaagcgggcaggctgcaaacgacgaaaactacgctttagtagcttaataactctgatagtgctagtgtagatctc

**C0062**	atgaaaaacataaatgccgacgacacatacagaataattaataaaattaaagcttgtagaagcaat<br>aatgatattaatcaatgcttatctgatatgactaaaatggtacattgtgaatattatttactcgcgatca<br>tttatcctcattctatggttaaatctgatatttcaatcctagataattaccctaaaaaatggaggcaata<br>ttatgatgacgctaatttaataaaatatgatcctatagtagattattctaactccaatcattcaccaatt<br>aattggaatatatttgaaaacaatgctgtaaataaaaaatctccaaatgtaattaaagaagcgaaaa<br>catcaggtcttatcactgggtttagtttccctattcatacggctaacaatggcttcggaatgcttagtttt<br>gcacattcagaaaaagacaactatatagatagtttatttttacatgcgtgtatgaacataccattaatt<br>gttccttctctagttgataattatcgaaaaataaatatagcaaataataaatcaaacaacgatttaacc<br>aaaagagaaaaagaatgtttagcgtgggcatgcgaaggaaaaagctcttgggatatttcaaaaata<br>ttaggttgcagtgagcgtactgtcactttccatttaaccaatgcgcaaatgaaactcaatacaacaaa<br>ccgctgccaaagtatttctaaagcaattttaacaggagcaattgattgcccatactttaaaaattaata<br>acactgatagtgctagtgtagatcac

**I0500**	ttatgacaacttgacggctacatcattcactttttcttcacaaccggcacggaactcgctcgggctggccccggtgcattttttaaatacccgcgagaaatagagttgatcgtcaaaaccaacattgcgaccgacggtggcgataggcatccgggtggtgctcaaaagcagcttcgcctggctgatacgttggtcctcgcgccagcttaagacgctaatccctaactgctggcggaaaagatgtgacagacgcgacggcgacaagcaaacatgctgtgcgacgctggcgatatcaaaattgctgtctgccaggtgatcgctgatgtactgacaagcctcgcgtacccgattatccatcggtggatggagcgactcgttaatcgcttccatgcgccgcagtaacaattgctcaagcagatttatcgccagcagctccgaatagcgcccttccccttgcccggcgttaatgatttgcccaaacaggtcgctgaaatgcggctggtgcgcttcatccgggcgaaagaaccccgtattggcaaatattgacggccagttaagccattcatgccagtaggcgcgcggacgaaagtaaacccactggtgataccattcgcgagcctccggatgacgaccgtagtgatgaatctctcctggcgggaacagcaaaatatcacccggtcggcaaacaaattctcgtccctgatttttcaccaccccctgaccgcgaatggtgagattgagaatataacctttcattcccagcggtcggtcgataaaaaaatcgagataaccgttggcctcaatcggcgttaaacccgccaccagatgggcattaaacgagtatcccggcagcaggggatcattttgcgcttcagccatacttttcatactcccgccattcagagaagaaaccaattgtccatattgcatcagacattgccgtcactgcgtcttttactggctcttctcgctaaccaaaccggtaaccccgcttattaaaagcattctgtaacaaagcgggaccaaagccatgacaaaaacgcgtaacaaaagtgtctataatcacggcagaaaagtccacattgattatttgcacggcgtcacactttgctatgccatagcatttttatccataagattagcggatcctacctgacgctttttatcgcaactctctactgtttctccatacccgtttttttgggctagc
**J23100**	ttgacggctagctcagtcctaggtacagtgctagc
**J23105**	tttacggctagctcagtcctaggtactatgctagc
**R0011**	aattgtgagcggataacaattgacattgtgagcggataacaagatactgagcaca
**R0062**	acctgtaggatcgtacaggtttacgcaagaaaatggtttgttatagtcgaataaa
**R0040**	tccctatcagtgatagagattgacatccctatcagtgatagagatactgagcac

**E1010**	atggcttcctccgaagacgttatcaaagagttcatgcgtttcaaagttcgtatggaaggttccgttaac<br>ggtcacgagttcgaaatcgaaggtgaaggtgaaggtcgtccgtacgaaggtacccagaccgctaaa<br>ctgaaagttaccaaaggtggtccgctgccgttcgcttgggacatcctgtccccgcagttccagtacgg<br>ttccaaagcttacgttaaacacccggctgacatcccggactacctgaaactgtccttcccggaaggttt<br>caaatgggaacgtgttatgaacttcgaagacggtggtgttgttaccgttacccaggactcctccctgc<br>aagacggtgagttcatctacaaagttaaactgcgtggtaccaacttcccgtccgacggtccggttatg<br>cagaaaaaaaccatgggttgggaagcttccaccgaacgtatgtacccggaagacggtgctctgaaa<br>ggtgaaatcaaaatgcgtctgaaactgaaagacggtggtcactacgacgctgaagttaaaaccacct<br>acatggctaaaaaaccggttcagctgccgggtgcttacaaaaccgacatcaaactggacatcacctc<br>ccacaacgaagactacaccatcgttgaacagtacgaacgtgctgaaggtcgtcactccaccggtgct<br>taataacgctgatagtgctagtgtagatcgc
**B0014**	tcacactggctcaccttcgggtgggcctttctgcgtttatatactagagagagaatataaaaagccag<br>attattaatccggcttttttattattt
**K176000**	acctgtaggatcgtacaggtttacgcaagaaaatggtttgttatagtcgaatatccctatcagtgatag<br>aga
**K914003**	ccacaattcagcaaattgtgaacatcatcacgttcatctttccctggttgccaatggcccattttcctgt<br>cagtaacgagaaggtcgcgtattcaggcgctttttagactggtcgtaatgaa
**K1420004**	ctacaccgcgtcggcaccacgcggctctttctccccttgcagcgaagcaatcagcgggcaagaaacg<br>ttcccttgccgcgcatggcaggcgaacacaagttcggatagcacggtttccatgcgcgccaggtcgg<br>tcatttttcgcgcacgtcctgaagcttgtgctcggccaggctgctggcttcctcgcagtgggtgccgtc<br>atccagcctcagcagctctgcgatctcgtcgaggctgaatccgagccgctgggctgatttcacgaagc<br>gcacccgcgtcacatccgcctcgccatagcggcggatgctgccatagggcttgtccggctccggcaa<br>caagcccttgcgctgatagaaccggattgtttccacgttgaccccggccgccttggcgaaaacgcca<br>atagtcagattctccaaattttttccat
**K346002**	ttccatatcgcttgactccgtacatgagtacggaagtaaggttacgctatccaatcc

Supplementary Table 2. Genetic parts sequence

Supplementary Figures

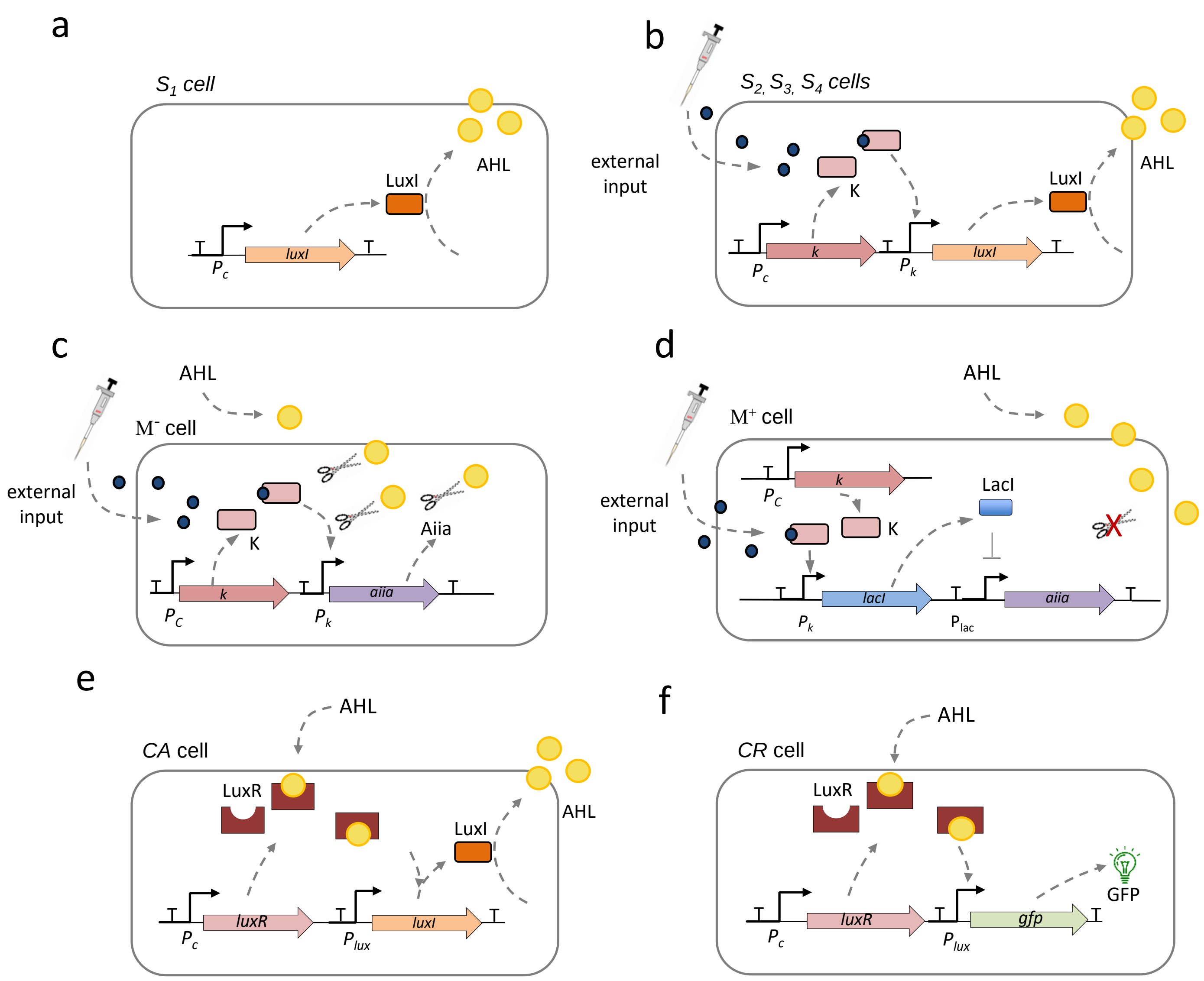

Supplementary Figure 1. Genetic architecture of the cell library. a. S_1 cells express LuxI downstream of the constitutive promoter P_C. LuxI generates AHL molecules which are secreted. **b.** In S_2, S_3 and S_4 cells, the expression of LuxI is regulated by an externally inducible promoter P_k (arabinose-inducible promoter P_{BAD} in S_2 cells, rhamnose-inducible promoter P_{rham} in S_3 cells and mercury-inducible promoter P_{mer} in cells S_4). K represents the receptor protein, AraC, RhaR and MerR respectively. **c.** In negative modulatory cells M^-, Aiia expression is regulated by an external inducible promoter P_k (P_{BAD}, Ptet and Pram respectively). The corresponding receptor proteins K are Arac, aTc and RhaS respectively. **d.** In positive modulatory cells M^+, the expression of LacI repressor is regulated by an external inducible promoter P_k (similar to negative modulatory cells) which in turns, negatively regulate Aiia expression. For b, c, and d, the external input (green circles) is either aTc, arabinose or rhamnose. **e.** Auto-amplifier cells (CA) express LuxI in the presence of AHL, which binds to and dimerises the LuxR transcription factor, that subsequently induces more AHL. **f.** Reporter cells (CR) produce GFP as a final output in the presence of AHL. Symbol T represent a double-terminator sequence.

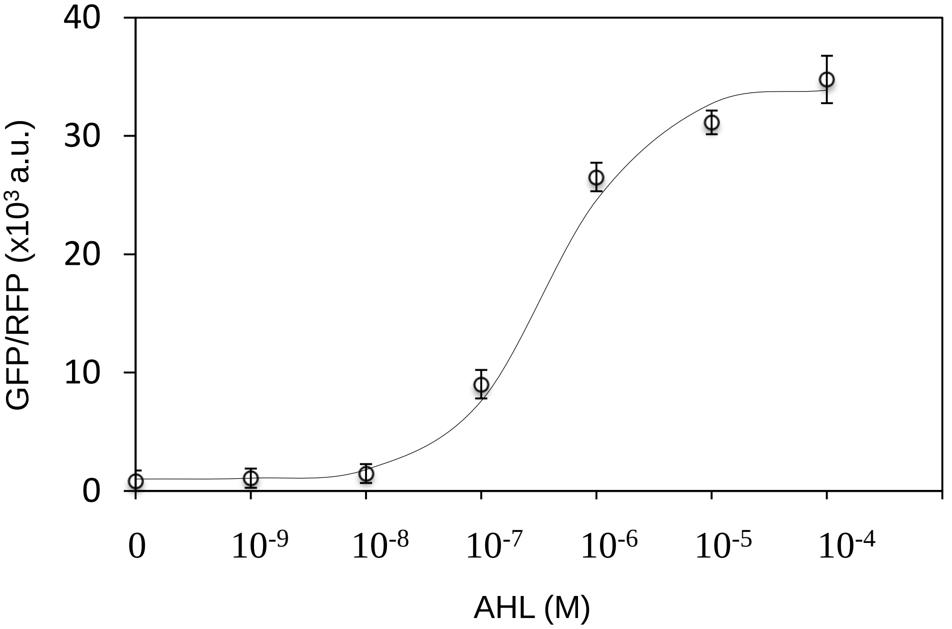

Supplementary Figure 2. AHL Transfer function of CR cells. Dots are the normalized GFP expression of CR cells grown on the paper surface at different AHL concentrations. The solid line indicates the mathematical fitting of the data, according to equation (S.1). Error bars are the standard deviation (SD) of three independent experiments. Data are presented as mean values +/- SD.

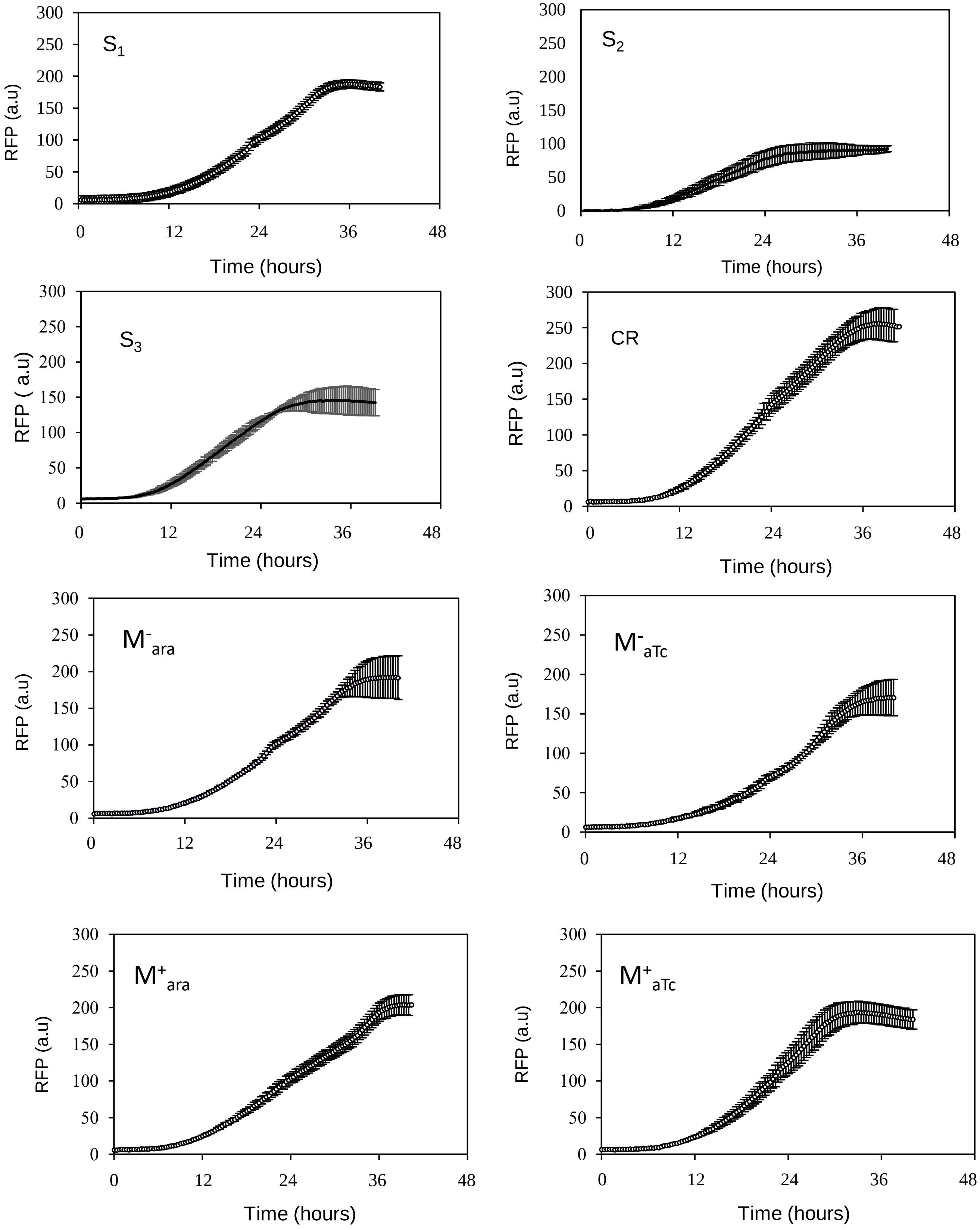

Supplementary Figure 3. **Cell growth.** Increase in red fluorescent protein (RFP) levels over time associated with cell growth in each indicated cell types. Error bars are the standard deviation (SD) from three independent measurements. Data are presented as mean values +/- SD.

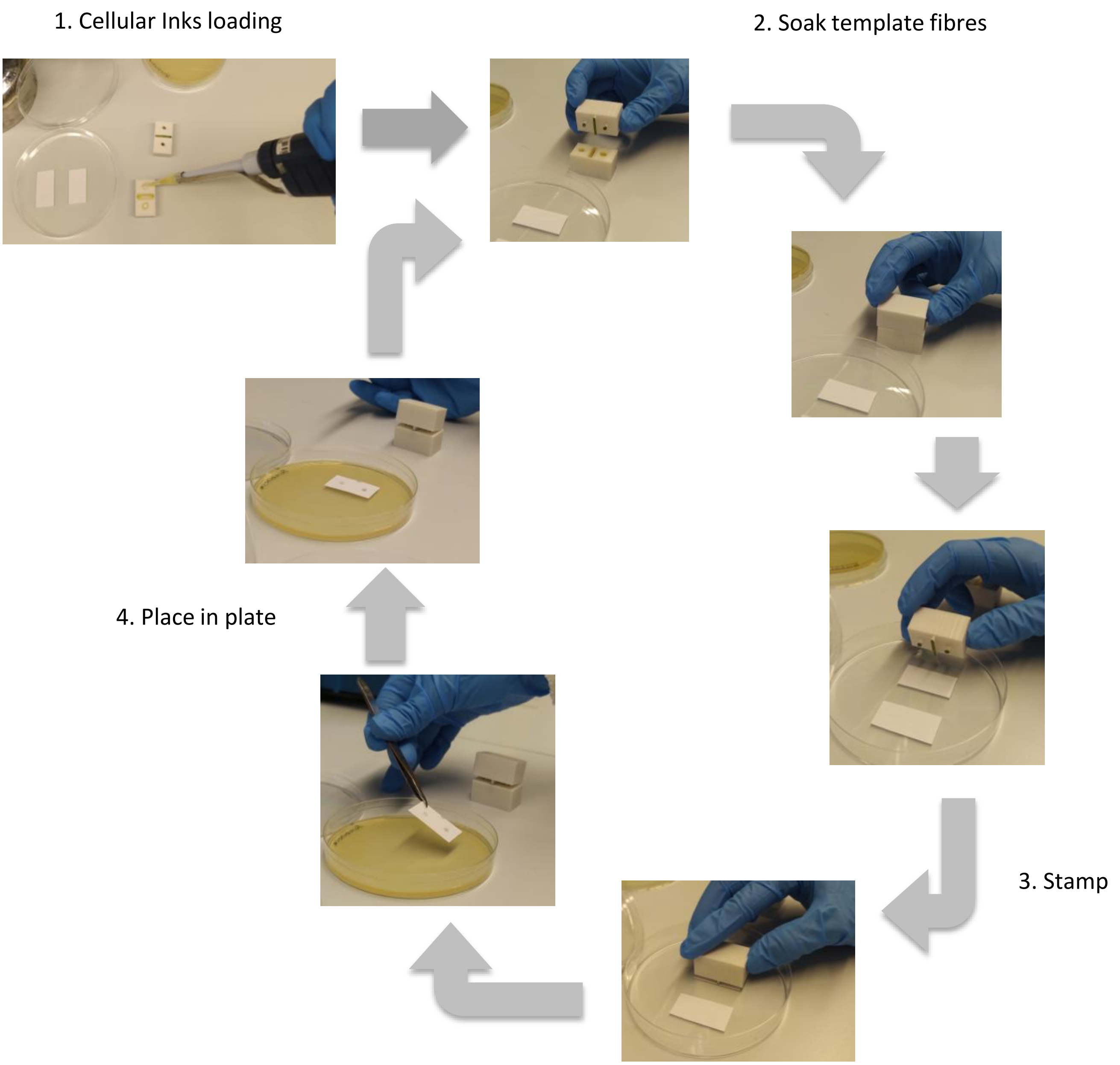

Supplementary Figure 4. Stamping process. Pictures visually describe the process made to stamp our circuits. Prototypes are filled with different cellular inks, the template is then soaked and the circuit is stamped in a paper strip. This strip is then placed on an LB-agar plate to incubate at 37ºC.

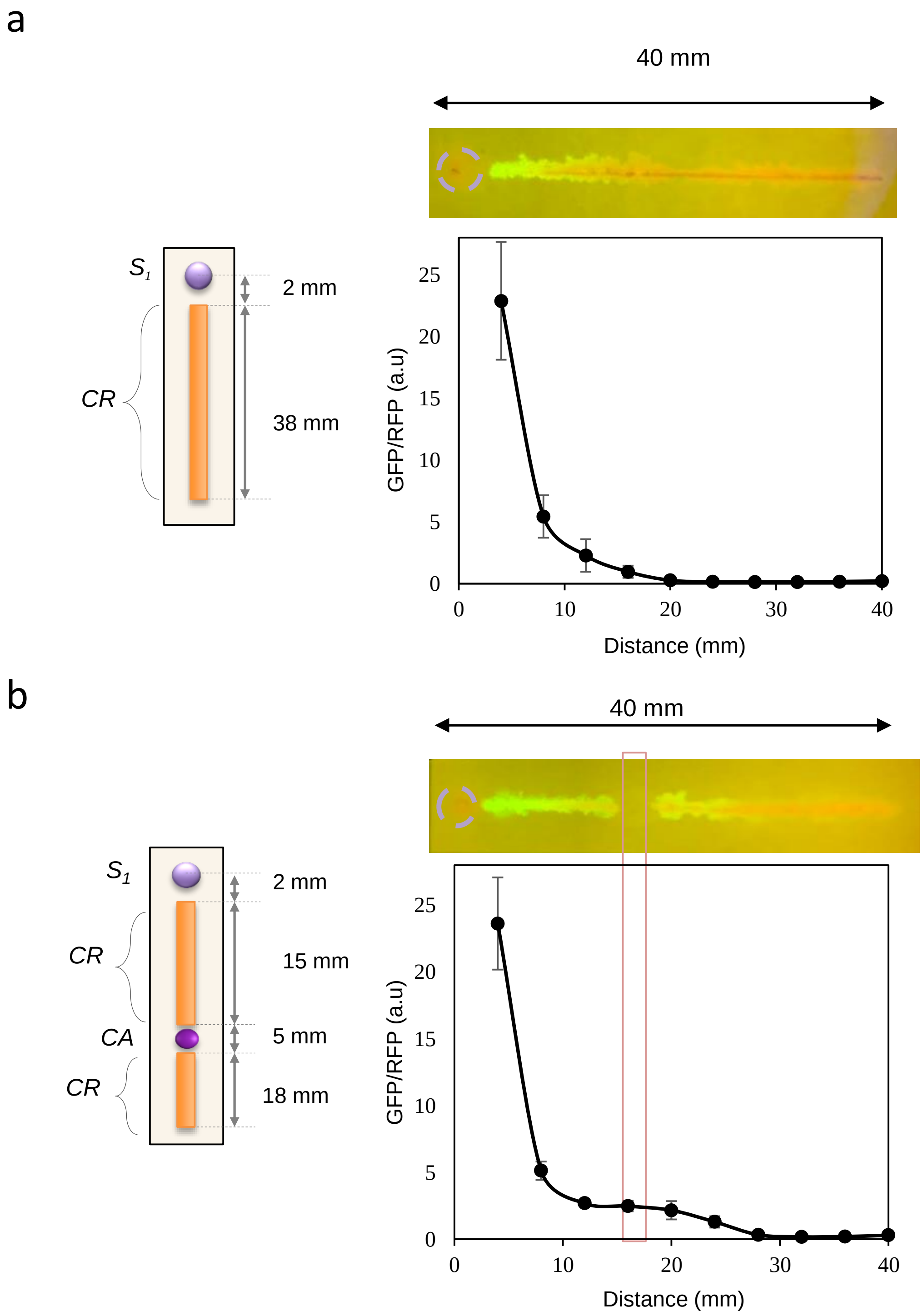

Supplementary Figure 5. AHL signalling through paper diffusion. **a, b,** Left. Schematic representation of the different cells used at each of the indicated distance points along the paper. S_1 cells, constitutively produce AHL; CR cells are GFP reporter cells; CA cells are auto-amplifier cells inserted to restore the signalling. **a, b,** Right. Numerical quantification of the GFP levels along each strip, acquired by scanning the surface of the paper after incubation during 24h at 37ºC. GFP levels are normalized by RFP, which correlates with cell population. Error bars are standard deviation (SD) of three independent experiments. Data are presented as mean values +/- SD.

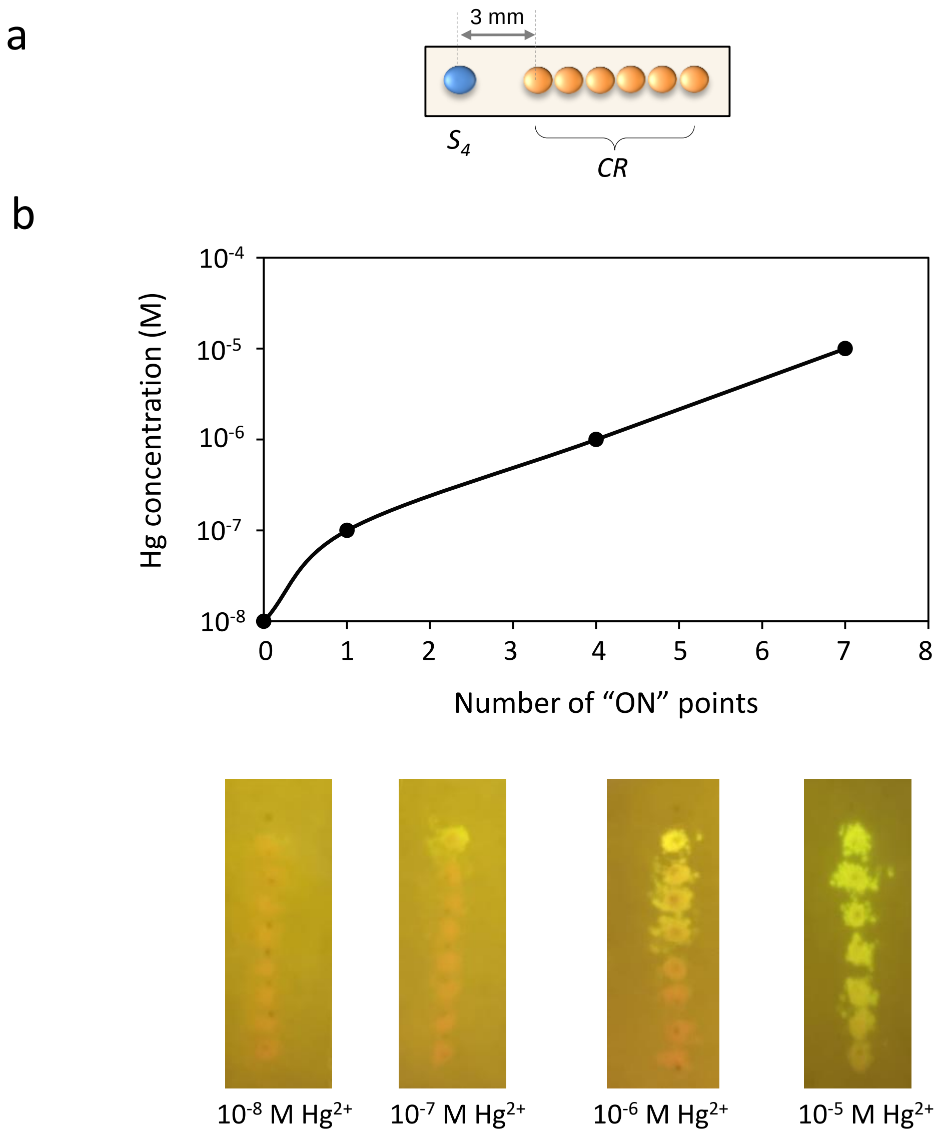

Supplementary Figure 6. Mercury biosensor prototype. **a.** Schematic representation of the different cells used for the biosensor assembly and characterization. In this case, S_4 cells are engineered cells which respond to different mercury concentrations and produce AHL molecules. The production of AHL is dependent on the mercury concentration, thus, the more mercury in the media, the more AHL signal is released. This signal sent is then detected by CR cells. **b.** Correlation between the CR points turned “ON” and the mercury concentration is displayed. As seen in the pictures, a visual quantification can be obtained by checking the number of CR cells “ON”, i.e. expressing GFP. Three independent experiments were performed from each mercury concentration and results are consistent.

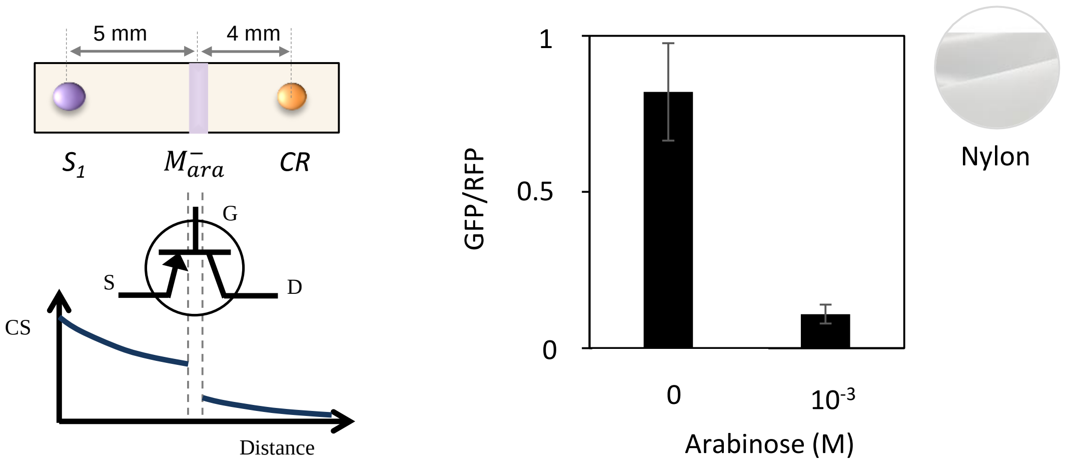

Supplementary Figure 7. **Transistor-like architecture printed on nylon fabric. a.** Mapping of a general transistor architecture on a cellular printed pattern composed of three main components: a source (S), a gate (G) that responds to external inputs and a drain (D) as the final output responding to the presence or absence of our carrying signal (CS). **b.** Circuit response. In the absence of external input, i.e. arabinose, the CS encoded in the production of AHL molecules by S_1 cells diffuses along the surface, inducing GFP expression in reporter cells CR. In the presence of 10^{-3} M arabinose (Ara), the modulatory element M^{-}_{ara} produces the AHL cleaving enzyme Aiia, which degrades the CS. Experiments are performed on a nylon fabric. Error bars are the standard deviation (SD) of three independent experiments. Data are presented as mean values +/- SD. The average fold change is 7.5x.

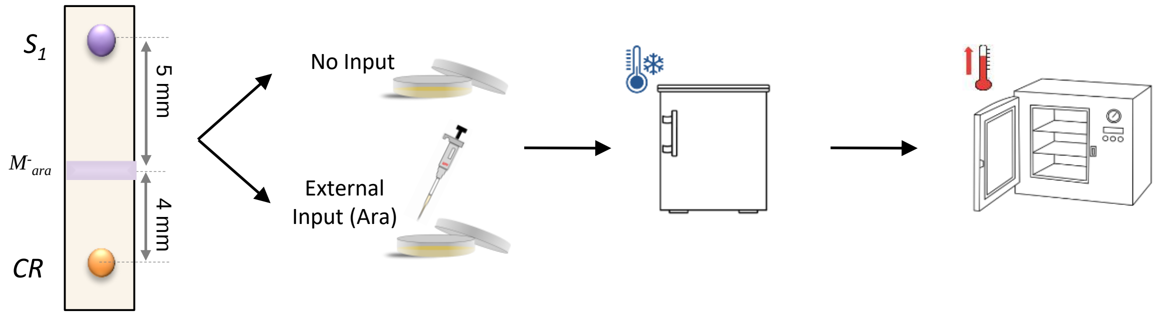

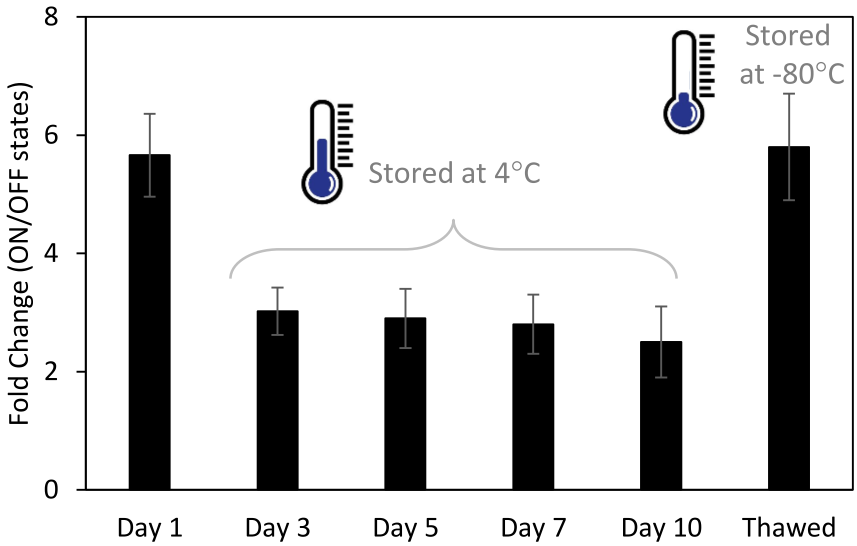

Supplementary Figure 8. Circuit stability and storage. Temporal stability of printed circuits. As a case study, a simple transistor-like device (identical to figure 1) was analysed. Circuits were stored on the fridge at 4°C. Every 2 days, circuits were grown at 37°C and measured after 24h. Despite the fold change reduces when circuits are stored in the fridge, differences between the ON/OFF states are still significant after 10 days. Circuits frozen at -80ºC do not show effects on their performance and maintain their functionality, once thawed. Error bars are the standard deviation (SD) of three independent experiments. Data are presented as mean values +/- SD.

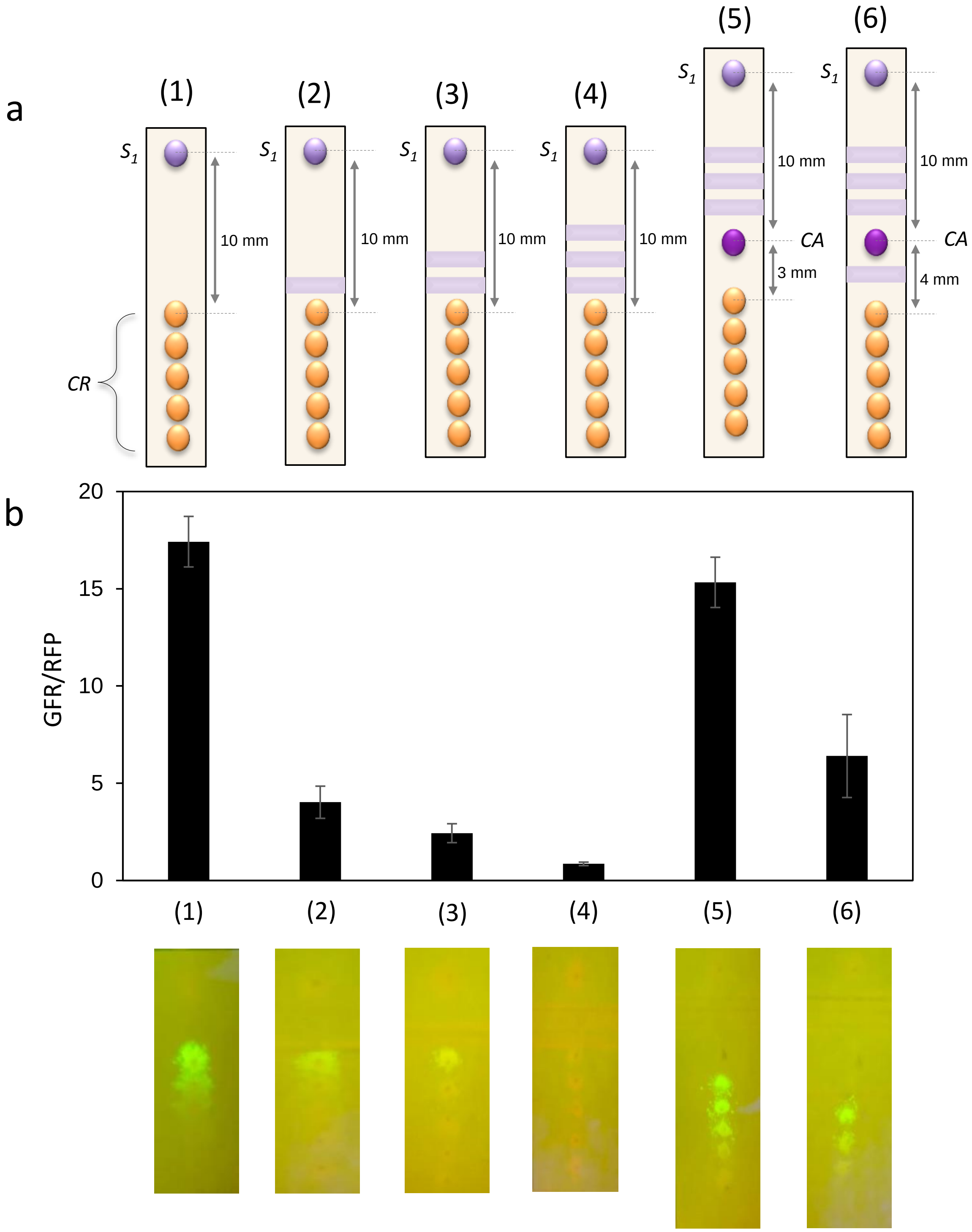

Supplementary Figure 9. AHL signalling diffusion decay through modulatory elements. Numerical quantification of the GFP acquired by scanning the surface of paper strips, with a schematic representation of the experimental setup. In order to determine the signal decay due to the presence of modulatory elements, different conditions were analysed four devices involving no modulators, 1 modulator, 2 modulators and 3 modulators. Additionally, auto-amplifier cells (CA) were added to re-establish the signal decay. **a.** Schematic representation of each cellular printed pattern with the corresponding distances. S_1 is located 10mm away from CR as it is the maximum distance used in all our experiments. ***b.*** Maximum GFP expression by reporter cells depending on the number of modulators. Error bars are the standard deviation (SD) of three independent experiments. Data are presented as mean values +/- SD. Several CR dots were printed to visualize the diffusion range of AHL molecules. GFP levels are normalized by RFP, which correlates with cell population. Below the chart, different pictures of the experiments can be shown.

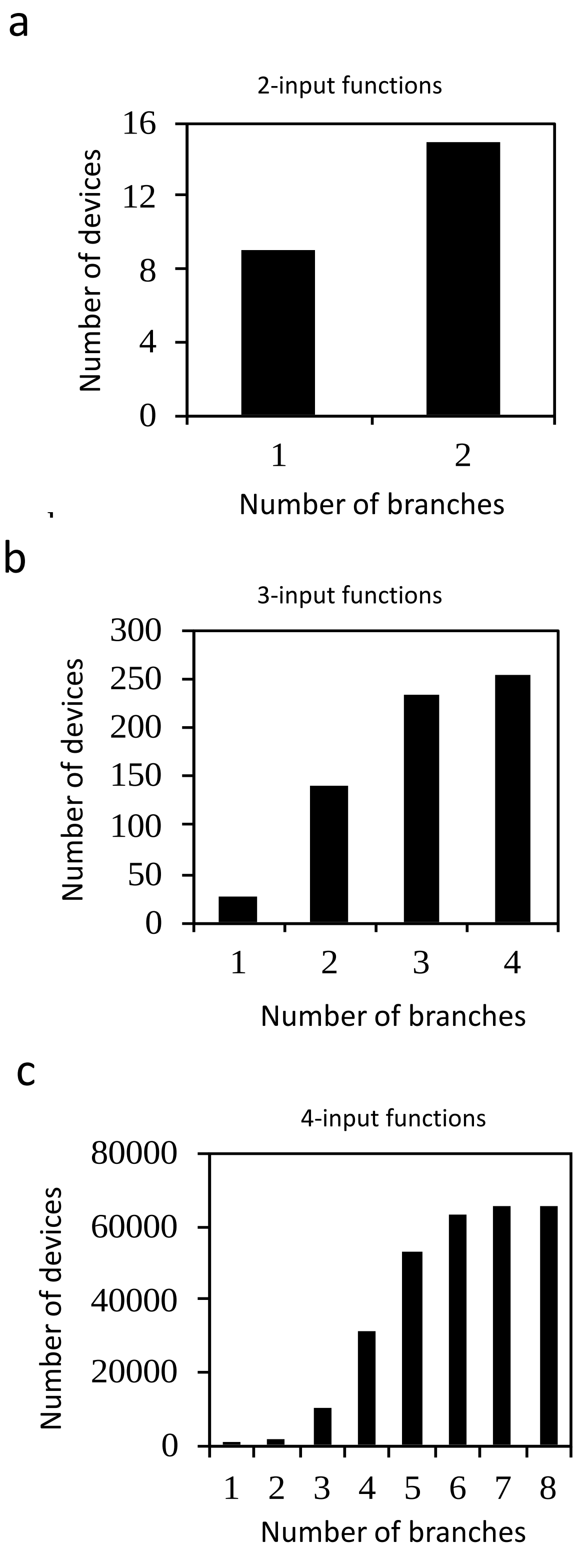

Supplementary Figure 10. Computational scalability. Dependence of the number of different devices versus the number of different branches required. **a**. Number of branches required to implement all logic circuits responding to 2 inputs. **b**. Number of branches required to implement all logic circuits responding to 3 inputs. **c**. Number of branches required to implement all logic circuits responding to 4 inputs.

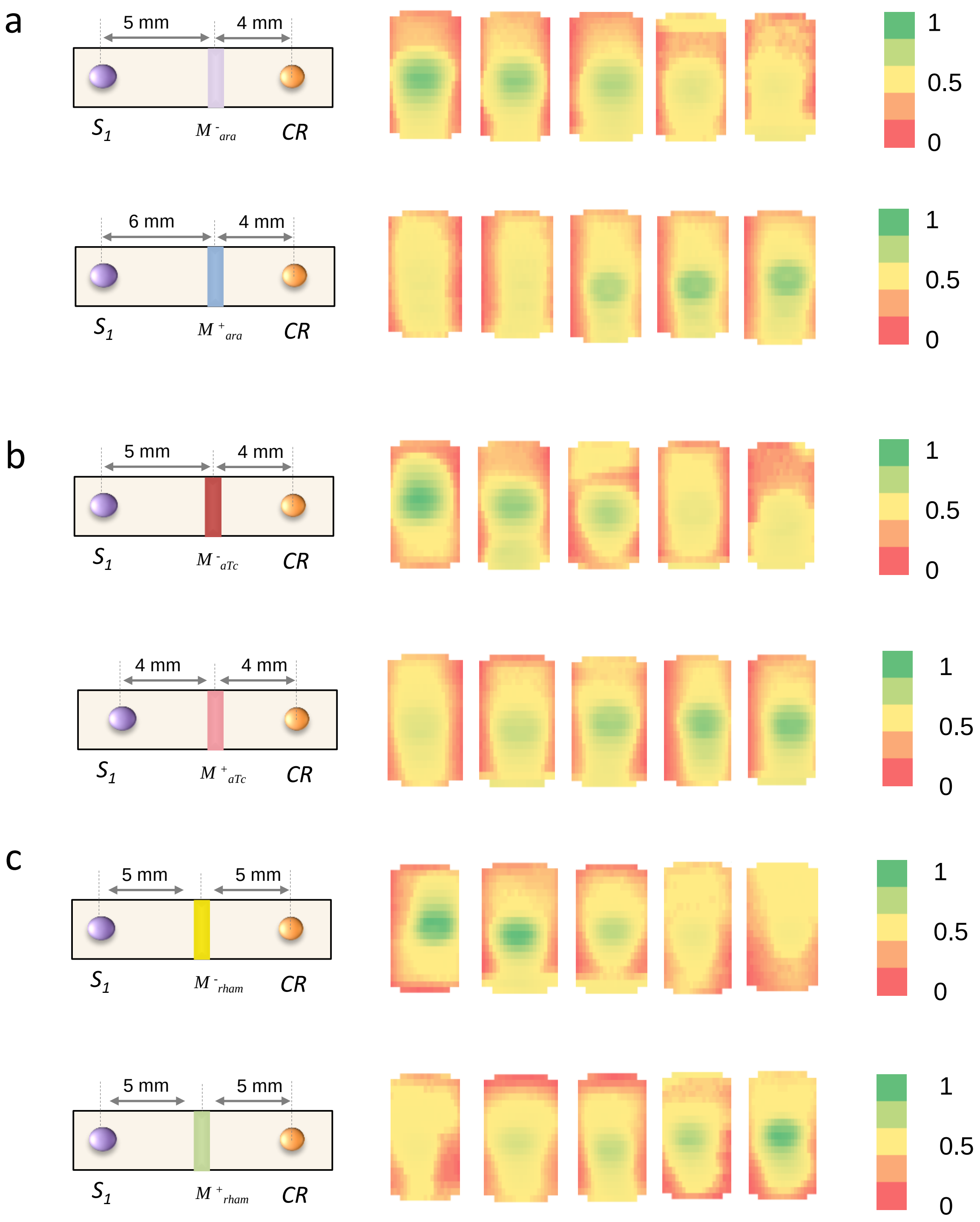

Supplementary Figure 11. Characterization of different modultatory cells. **a**. Normalized GFP for positive (M^{+}_{ara}) and negative (M^{-}_{ara}) modulations in response to arabinose. **b**. Normalized GFP for positive (M^{+}_{aTc}) and negative (M^{-}_{aTc}) modulations in response to aTc. **c**. Normalized GFP for positive (M^{+}_{rham}) and negative (M^{-}_{rham}) modulations in response to rhamnose.

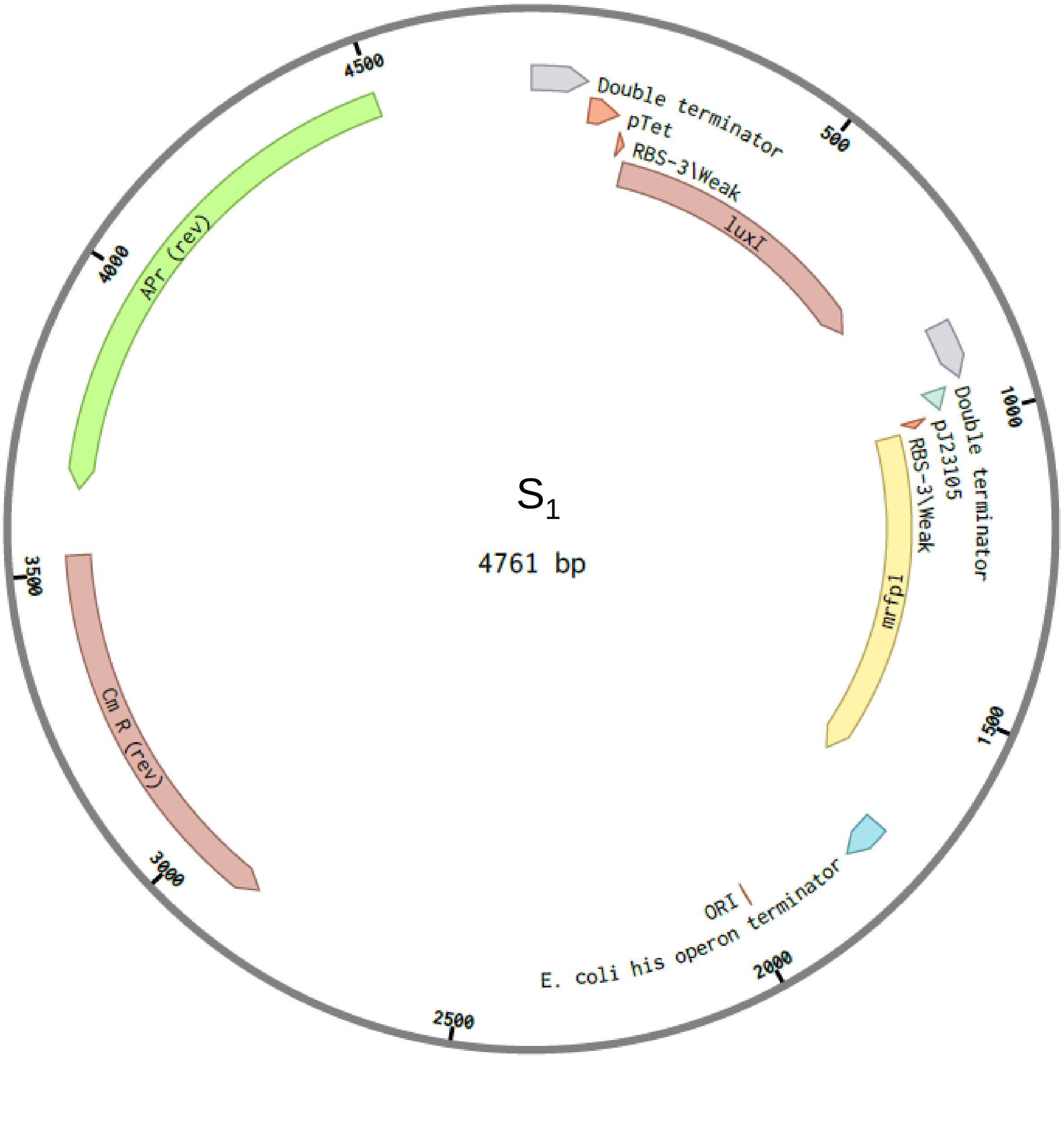

Supplementary Figure 12. Plasmid map of S_1 cells

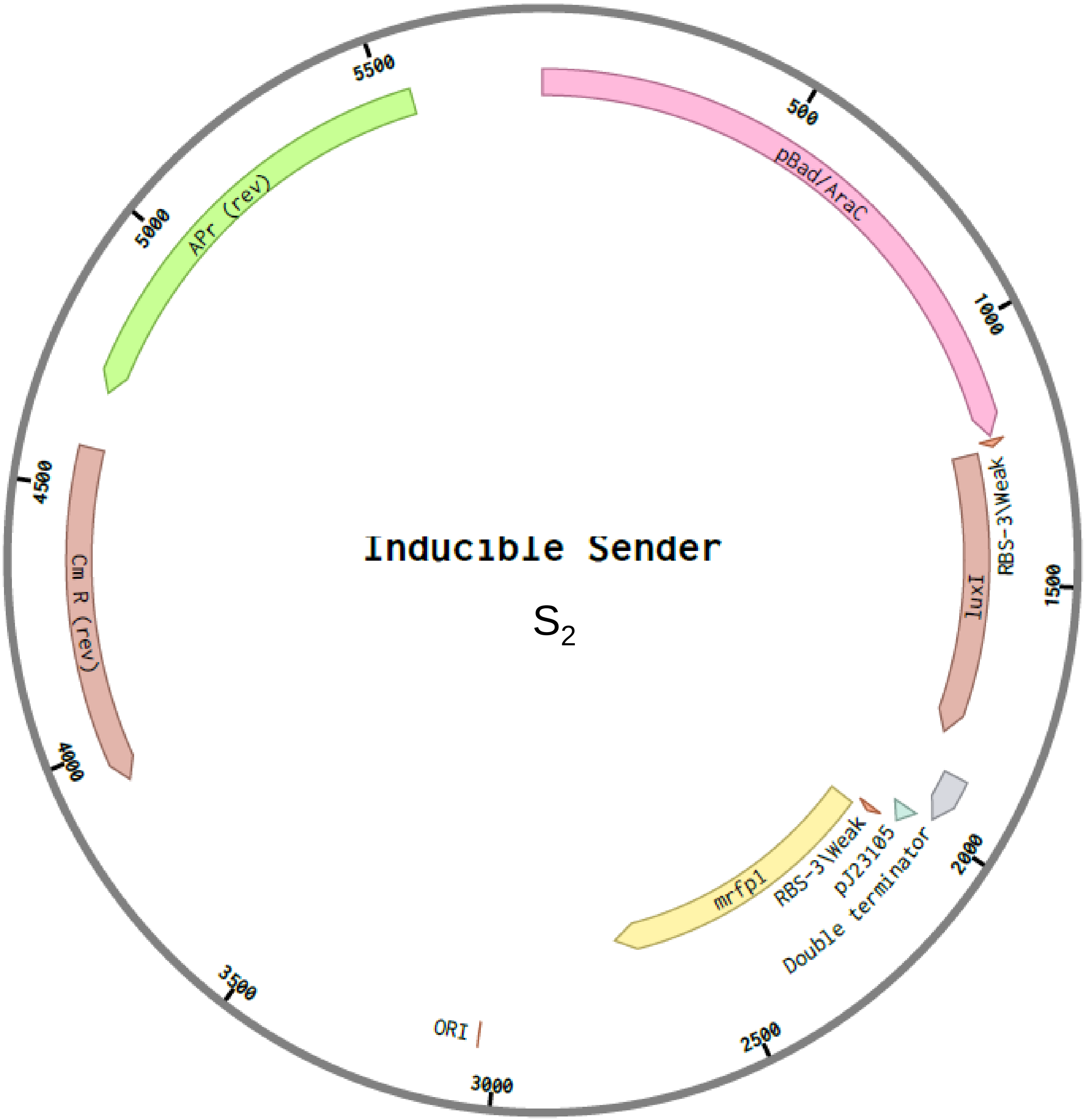

Supplementary Figure 13. Plasmid map of S_2 cells

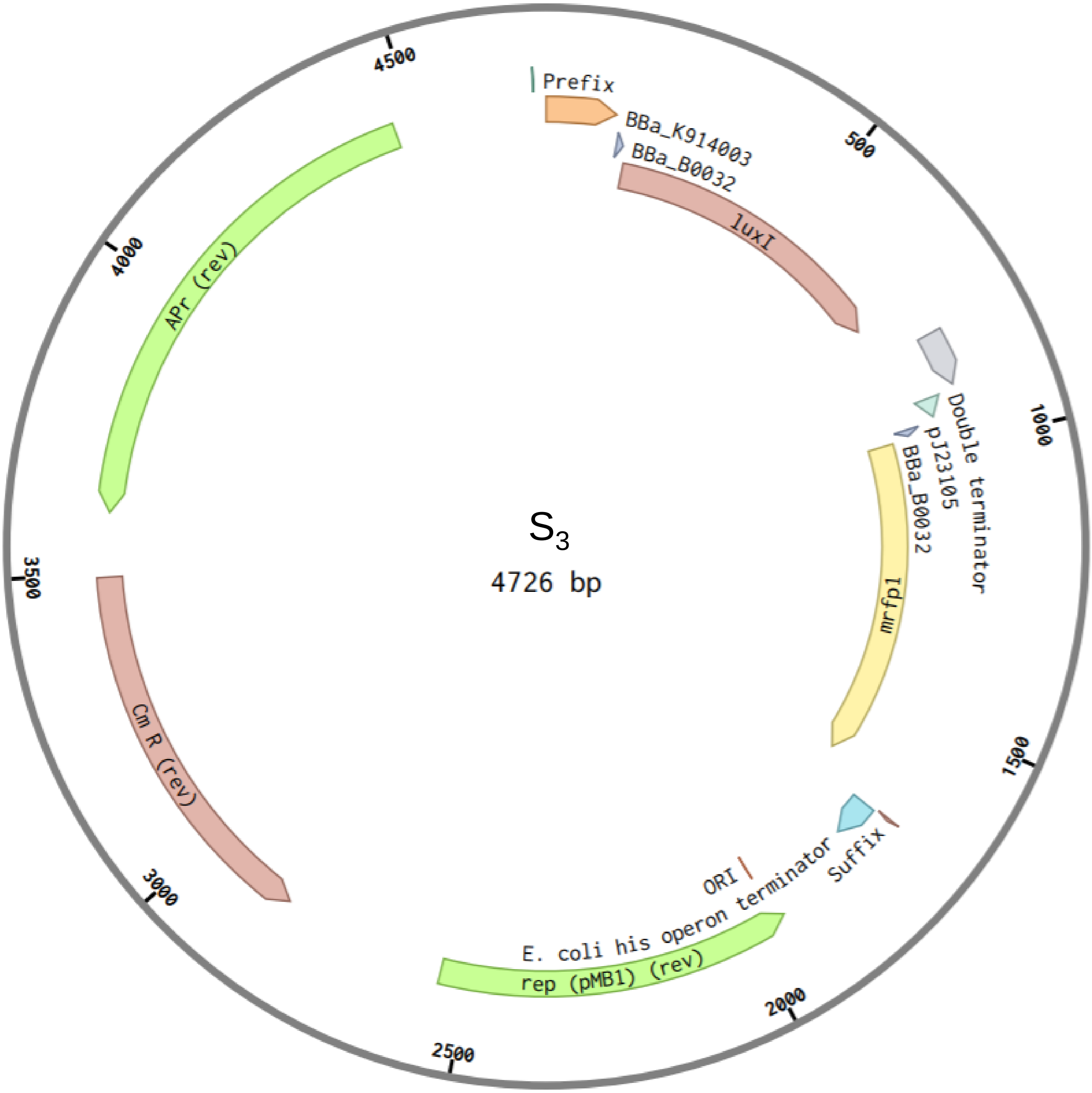

Supplementary Figure 14. Plasmid map of S_3 cells

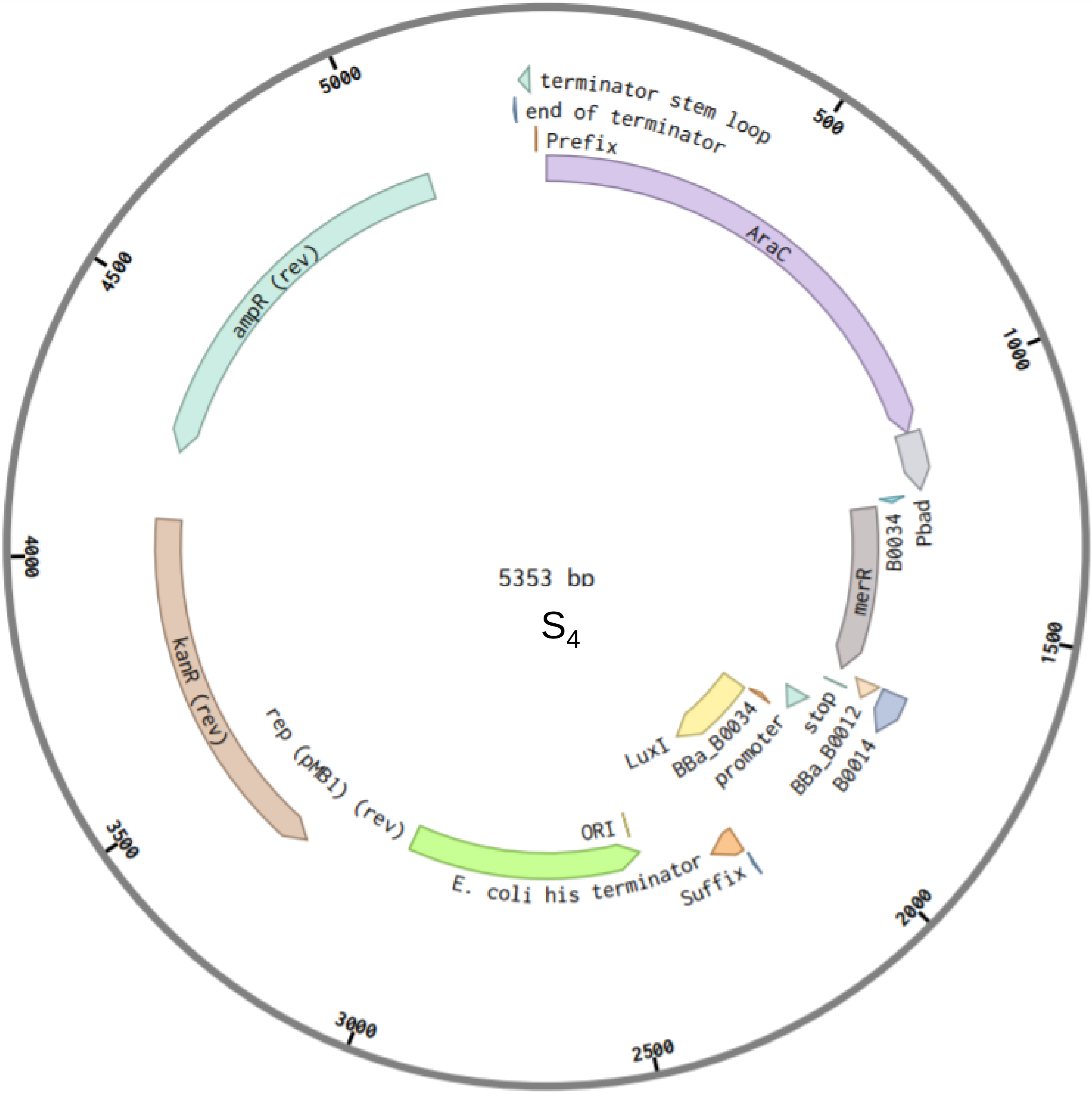

Supplementary Figure 15. Plasmid map of S_4 cells

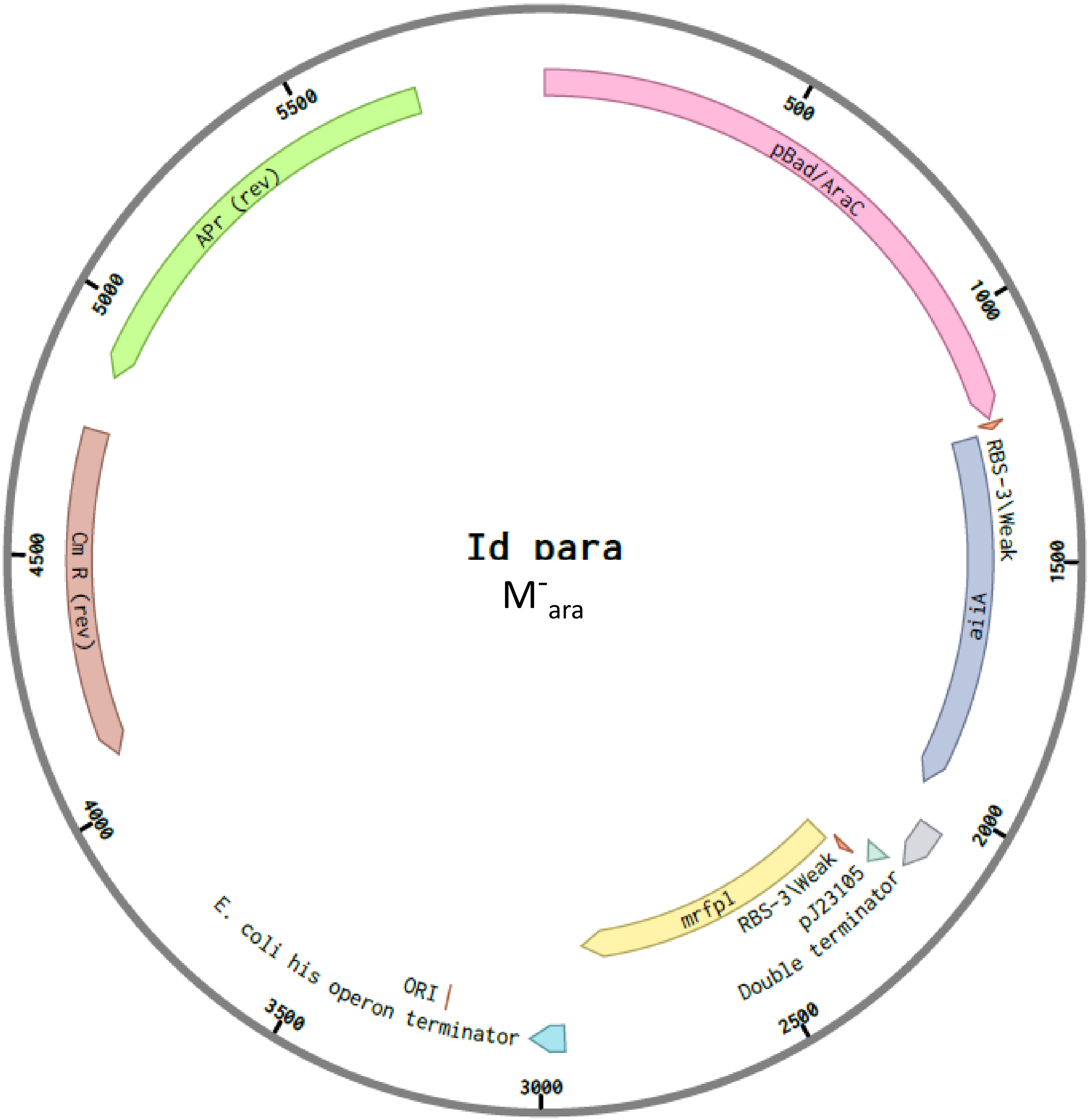

Supplementary Figure 16. Plasmid map of M^{-}_{ara} cells

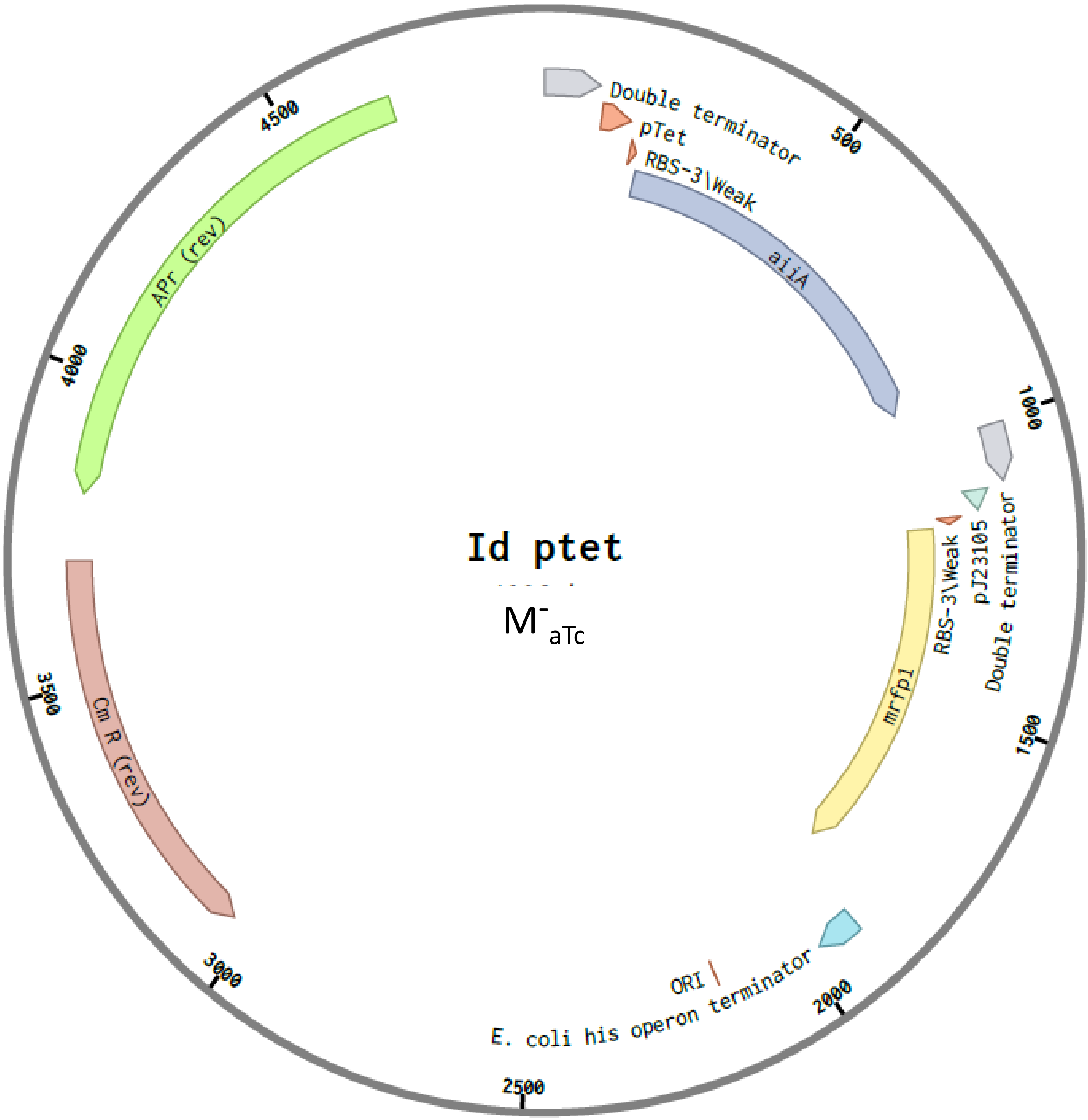

Supplementary Figure 17. Plasmid map of M$^{-}_{aTc}$ cells

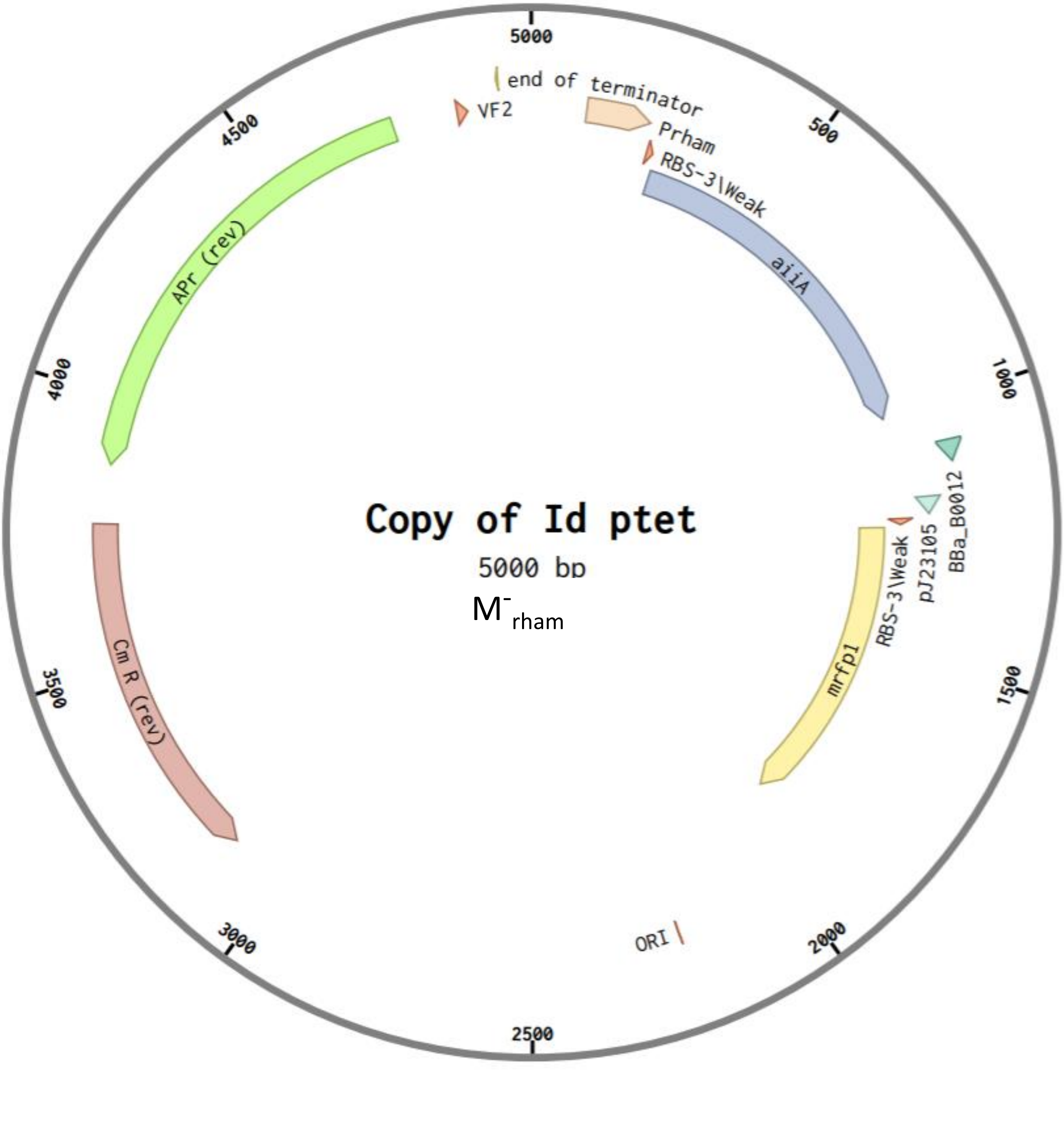

Supplementary Figure 18. Plasmid map of M^{-}_{rham} cells

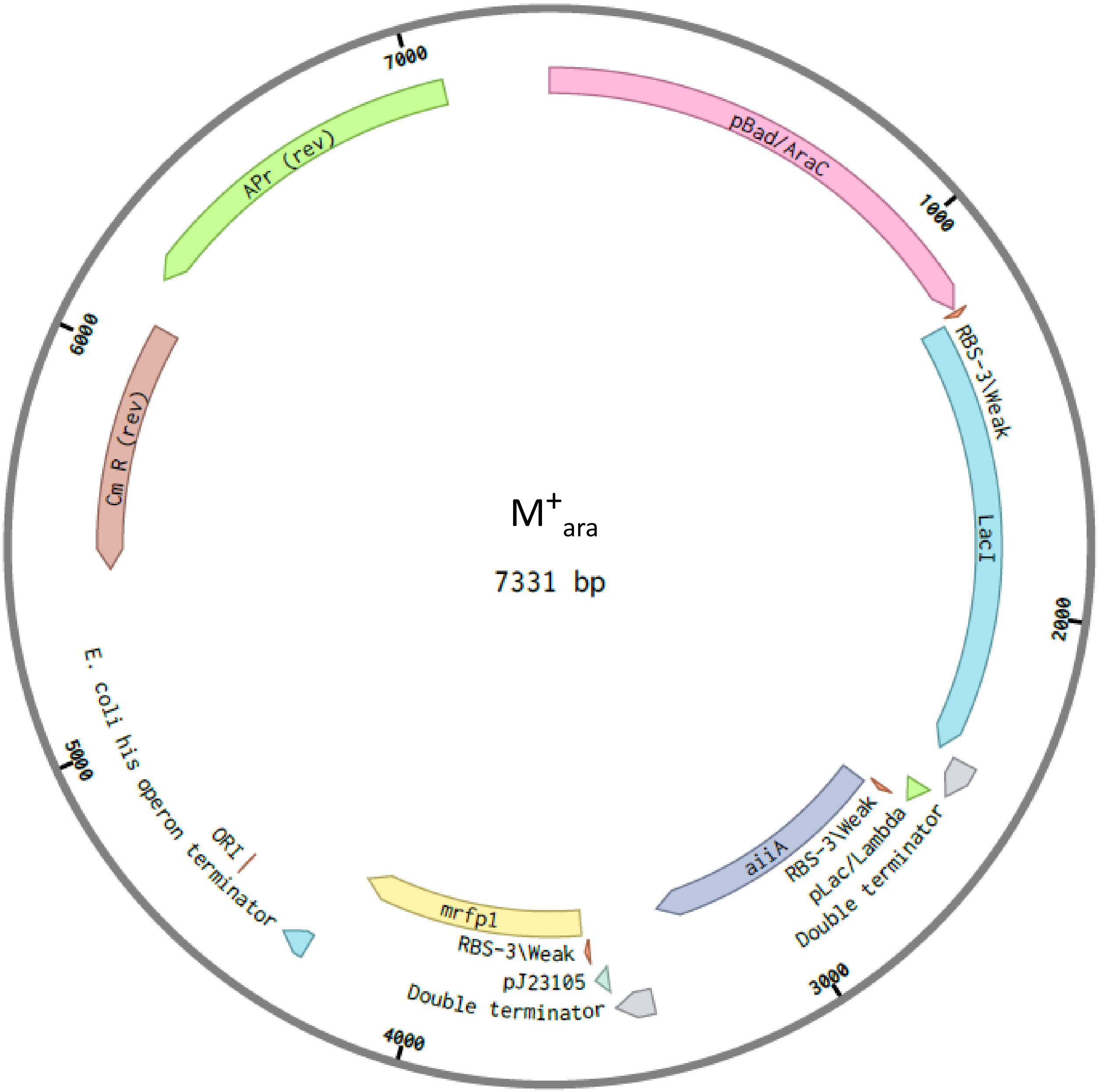

Supplementary Figure 19. Plasmid map of M^{+}_{ara} cells

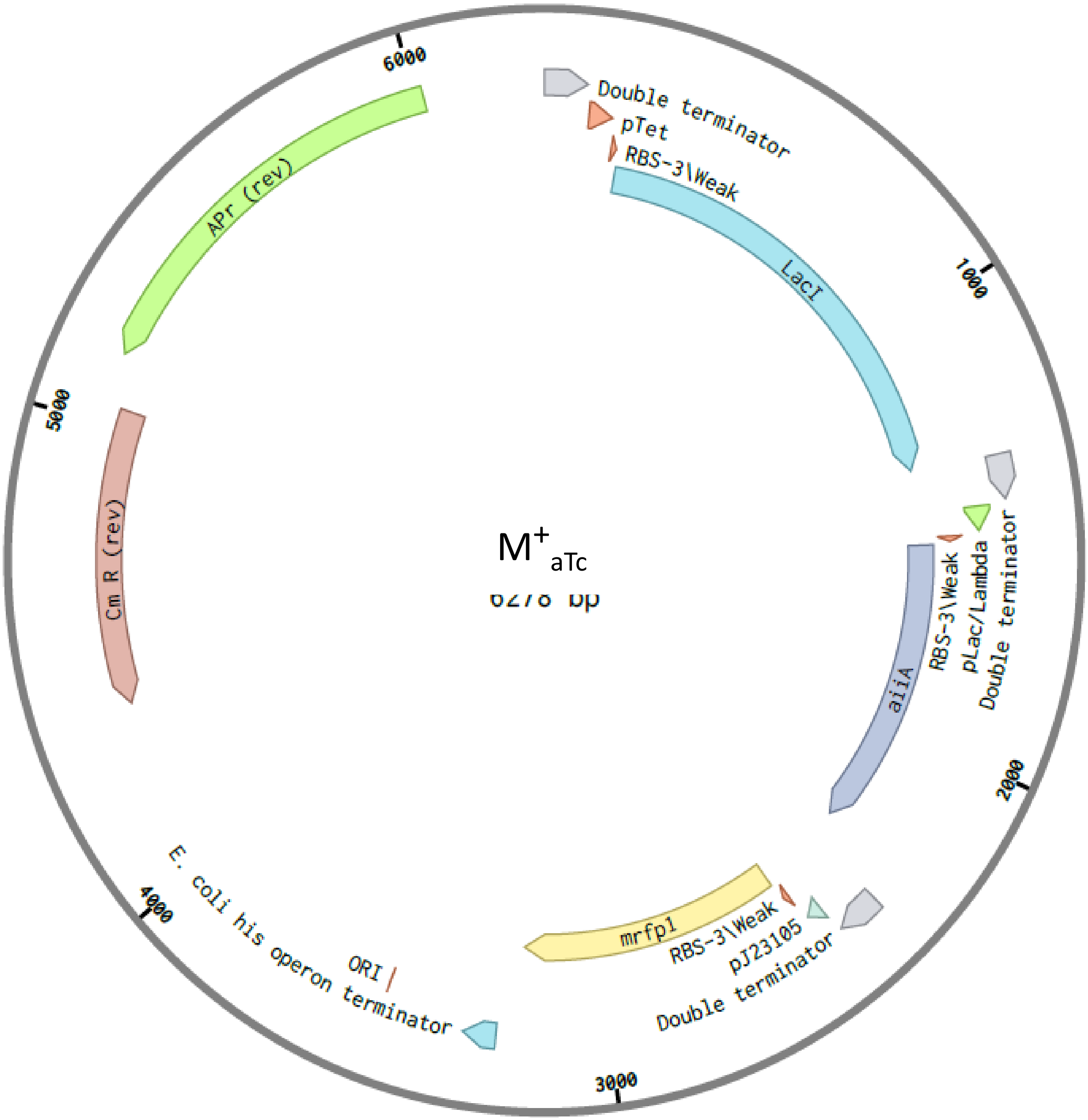

Supplementary Figure 20. Plasmid map of M^{+}_{aTc} cells

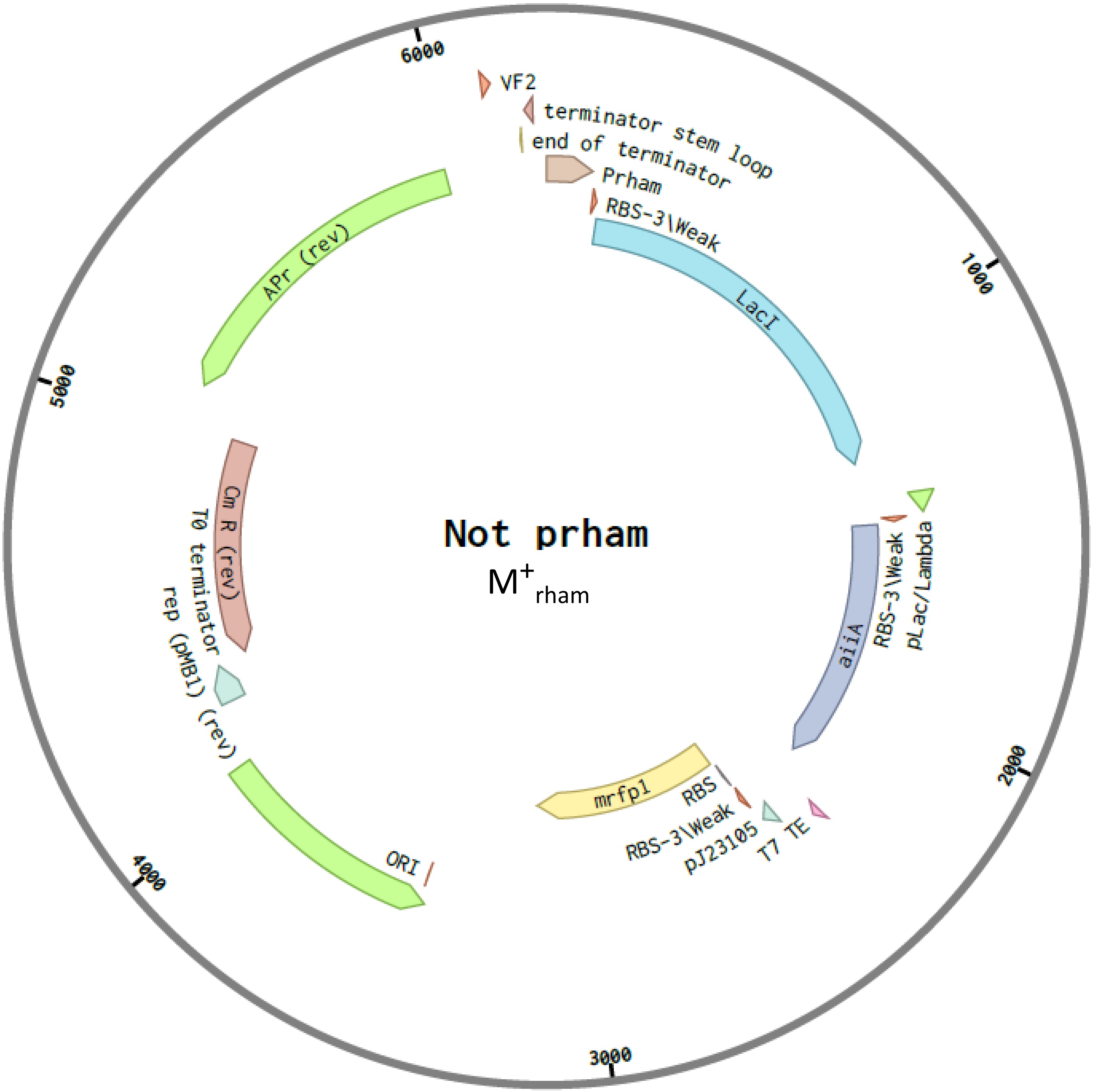

Supplementary Figure 21. Plasmid map of M^{+}_{rham} cells

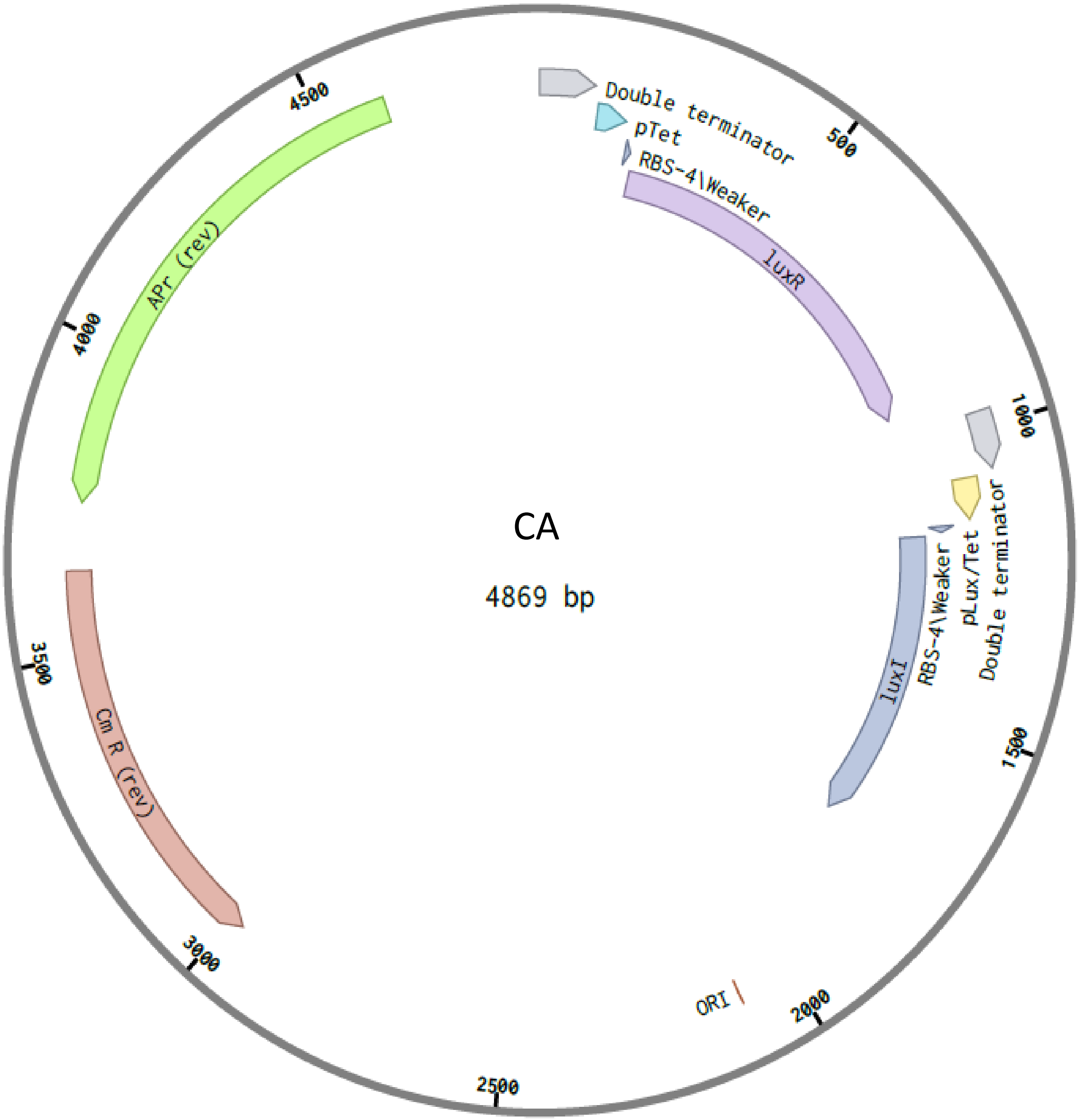

Supplementary Figure 22. Plasmid map of CA cells

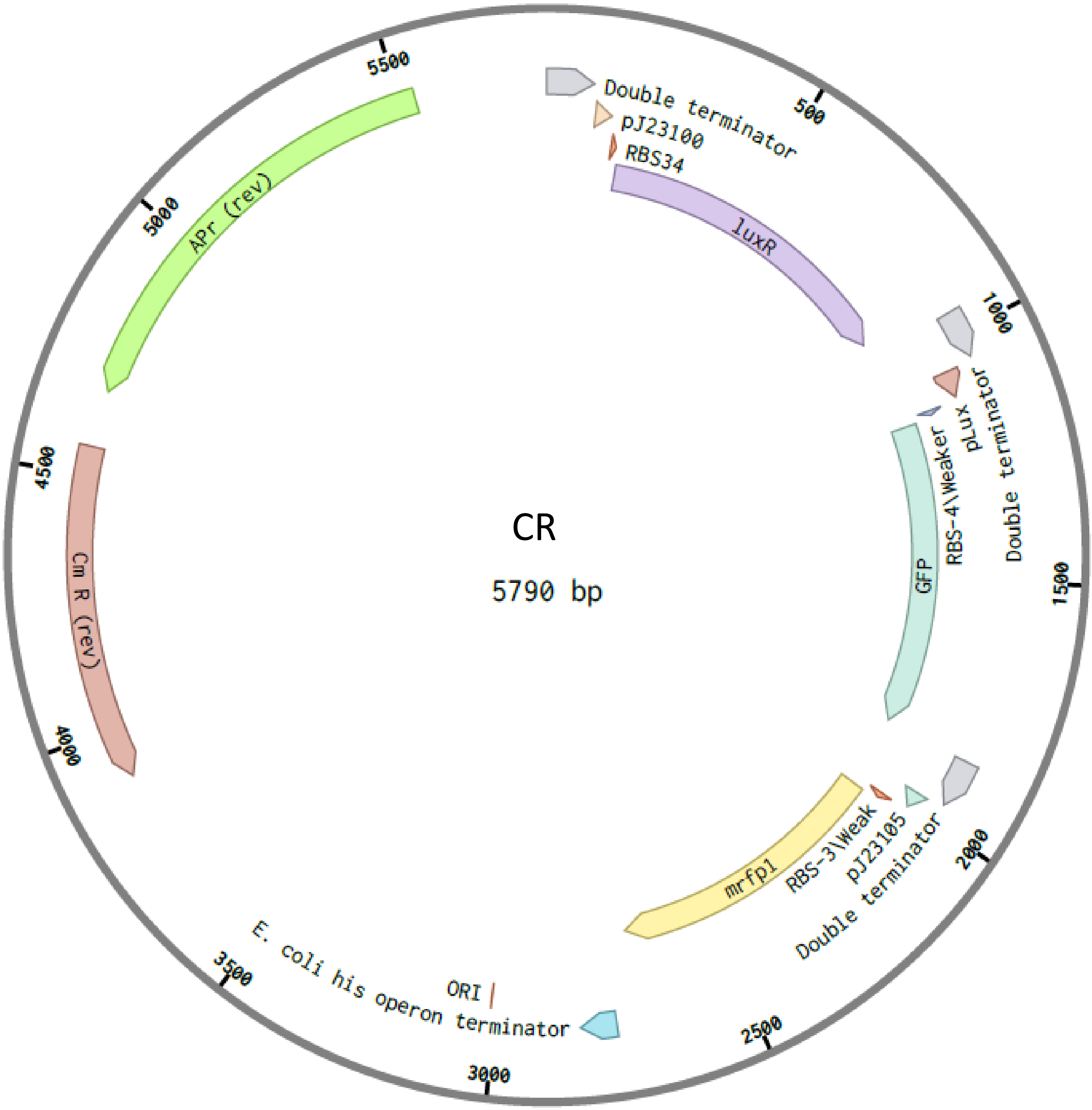

Supplementary Figure 23. Plasmid map of CR cells

Supplementary References

1. Fuqua, C., Winans, S. C. & Greenberg, E. P. Census and consensus in bacterial ecosystems:: The LuxR-LuxI Family of Quorum-Sensing Transcriptional Regulators. *Annu. Rev. Microbiol.* **50**, 727–751 (1996).
2. Schleif, R. AraC protein, regulation of the l-arabinose operon in Escherichia coli, and the light switch mechanism of AraC action. *FEMS Microbiol. Rev.* **34**, 779–796 (2010).
3. Georgi, C., Buerger, J., Hillen, W. & Berens, C. Promoter strength driving TetR determines the regulatory properties of tet-controlled expression systems. *PLoS One* **7**, (2012).
4. Pérez, P. D. & Hagen, S. J. Heterogeneous response to a quorum-sensing signal in the luminescence of individual vibrio fischeri. *PLoS One* **5**, (2010).
